# Development of a 3D functional assay and identification of biomarkers, predictive for response of high-grade serous ovarian cancer (HGSOC) patients to poly-ADP ribose polymerase inhibitors (PARPis): targeted therapy

**DOI:** 10.1186/s12967-020-02613-4

**Published:** 2020-11-19

**Authors:** Razan Sheta, Magdalena Bachvarova, Marie Plante, Marie-Claude Renaud, Alexandra Sebastianelli, Jean Gregoire, Jamilet Miranda Navarro, Ricardo Bringas Perez, Jean-Yves Masson, Dimcho Bachvarov

**Affiliations:** 1 Department of Molecular Medicine, Université Laval, Québec, QC G1V 0A6 Canada; 2 Centre de recherche du CHU de Québec, Oncology division, L’Hôtel-Dieu de Québec, 9 rue McMahon, Québec, QC G1R 3S3 Canada; 3 Department of Obstetrics and Gynecology, Université Laval, Québec, QC G1V 0A6 Canada; 4 Bioinformatics Department, Center for Genetic Engineering and Biotechnology, 10600 Havana, CP Cuba; 5 Department of Molecular Biology, Medical Biochemistry, and Pathology, Laval University Cancer Research Center, Québec, QC G1V 0A6 Canada

**Keywords:** High-grade serous ovarian cancer, PARP inhibitors homologous recombination repair pathway, Functional assay, Ascites, Primary cell cultures, 3D cellular model, Spheroids, Epithelial-to-mesenchymal transition, Biomarkers

## Abstract

**Background:**

Poly(ADP-ribose) polymerase inhibitors (PARPis) specifically target homologous recombination deficiency (HRD) cells and display good therapeutic effect in women with advanced-stage BRCA1/2-mutated breast and epithelial ovarian cancer (EOC). However, about 50% of high grade serous ovarian cancers (HGSOC) present with HRD due to epigenetic BRCA1 inactivation, as well as genetic/epigenetic inactivation(s) of other HR genes, a feature known as “BRCAness”. Therefore, there is a potential for extending the use of PARPis to these patients if HR status can be identified.

**Methods:**

We have developed a 3D (spheroid) functional assay to assess the sensitivity of two PARPis (niraparib and olaparib) in ascites-derived primary cell cultures (AsPCs) from HGSOC patients. A method for AsPCs preparation was established based on a matrix (agarose), allowing for easy isolation and successive propagation of monolayer and 3D AsPCs. Based on this method, we performed cytotoxicity assays on 42 AsPCs grown both as monolayers and spheroids.

**Results:**

The response to PARPis treatment in monolayer AsPCs, was significantly higher, compared to 3D AsPCs, as 88% and 52% of the monolayer AsPCs displayed sensitivity to niraparib and olaparib respectively, while 66% of the 3D AsPCs were sensitive to niraparib and 38% to olaparib, the latter being more consistent with previous estimates of HRD (40%–60%) in EOC. Moreover, niraparib displayed a significantly stronger cytotoxic effect in both in 3D and monolayer AsPCs, which was confirmed by consecutive analyses of the HR pathway activity (γH2AX foci formation) in PARPis-sensitive and resistant AsPCs. Global gene expression comparison of 6 PARPi-resistant and 6 PARPi-sensitive 3D AsPCs was indicative for the predominant downregulation of numerous genes and networks with previously demonstrated roles in EOC chemoresistance, suggesting that the PARPis-sensitive AsPCs could display enhanced sensitivity to other chemotherapeutic drugs, commonly applied in cancer management. Microarray data validation identified 24 potential gene biomarkers associated with PARPis sensitivity. The differential expression of 7 selected biomarkers was consecutively confirmed by immunohistochemistry in matched EOC tumor samples.

**Conclusion:**

The application of this assay and the potential biomarkers with possible predictive significance to PARPis therapy of EOC patients now need testing in the setting of a clinical trial.

## Background

Epithelial ovarian cancer (EOC) accounts for 4% of all cancers in women and is the leading cause of death from gynecologic malignancies, mainly due to its asymptomatic nature and the resulting lack of early diagnosis [1, 2]. EOC is histologically classified into different carcinoma subtypes, including low-grade serous, high-grade serous, endometrioid, mucinous and clear cell [3, 4]. Among these, high-grade serous ovarian carcinoma (HGSOC) represents the most frequent type, comprising about 70% of all advanced EOCs [4]. The standard treatment for EOC is debulking surgery followed by chemotherapy, usually platinum/taxane based. Although overall initial response rates are high, resistance to chemotherapy often develops, and only 10–15% of EOC patients achieve and maintain complete response to therapy [1]. Thus, the molecular pathogenesis of EOC is heterogeneous, and is reflected in the variability of clinical characteristics such as histological type, differentiation, potential for invasion and metastasis, and response to therapy and outcome, as there is an unmet need to improve treatment strategies for this deadly disease [5].

Approximately 20–25% of HGSOC are associated with germline and somatic mutations in one of the two cancer susceptibility genes, BRCA1 or BRCA2 [6, 7]. Other DNA damage signaling and repair genes such as CHK2, PALB2, FANCM, BRIP1, RAD51C and D, also contribute to EOC etiology [8]. Recognizing that inherited BRCA1/2 mutations are implicated in the cause of disease in a proportion of EOC patients, has led to the identification of the role of these genes in DNA repair. BRCA1 and BRCA2 are key components of the homologous recombination (HR) repair pathway; tumors with mutations in BRCA1/2 or other homologous recombination deficiency (HRD) genes are particularly sensitive to PARP inhibitors (PARPis)—a process called “synthetic lethality” [9, 10]. To date, three PARPis—olaparib, niraparib and rucaparib, have been approved by the Food and Drug Administration (FDA) and the European Medicines Agency (EMA) for treatment of EOC patients displaying complete or partial response to conventional platinum-based chemotherapy [11]. These drugs are currently investigated as single-agent, and as post-platinum maintenance therapy in numerous clinical trials enrolling EOC patients (recently reviewed in [12]). Additional candidates, such as veliparib, talazoparib, and iniparib, are currently being evaluated in preclinical studies and clinical trials [13]. Initially, women with advanced-stage BRCA1/2-mutated EOC were the subjects to treatment with PARPis [14]. However, it quickly became clear that BRCA1/2 mutations might not be the only markers for identifying EOC patients who can profit from PARPis therapy [15]. Indeed, a high proportion (up to 50%) of HGSOC cases present with HRD, due to epigenetic BRCA1 inactivation, as well as genetic/epigenetic inactivation(s) of other important HR genes (e.g. RAD51C, RAD51D, BRIP1, PALB2, BARD1 the MMR genes, and others), a feature known as “BRCAness” [16, 17]. Thus, an accurate estimate of HRD is likely to be a better predictor for response to PARPis than BRCA1/2 mutation status. A number of reports presented data on somatic BRCA1/2 and HR genes mutations in EOC [18–21], highlighting that both germline and somatic mutation analyses are quite essential for improving PARPis therapy. Moreover, it was demonstrated that PARPis can display therapeutic effects on HGSOC patients with platinum-sensitive/responsive disease, despite the lack of germline BRCA1/2 mutations [22]. Thus, patients with germline or somatic HRD will likely be suitable candidates for PARPis therapy; however, a major challenge facing the use of PARPis is the paucity of functional assays and/or biomarkers to identify EOC patients who may benefit from these agents [23, 24]. Different combinations of biomarkers with relevant clinicopathological features have been tested with a focus on detecting HRD cases that can profit from PARPis treatment [25]. Recently, next generation sequencing (NGS) assays that can detect HRD, as well as genome-wide loss of heterozygosity (LOH), telomeric allelic imbalance (TAI) and large-scale state transitions (LST), have been developed and evaluated in clinical trials [26, 27]. Two such assays, the ‘FoundationFocus CDx BRCA LOH’, [28], and the ‘myChoice HRD’ (Myriad; simultaneously analyzing LOH, TAI, and LST events) [29], have been FDA-approved for HRD diagnostics in EOC [30]. The predictive value of these assays were tested in randomized trials using niraparib and rucaparib treatment in EOC [12]; however, both assays were not able to accurately predict PARPis sensitivity in patients with relapsed, platinum-sensitive HGSOC [30]. Thus, and despite these efforts, there is currently no standard method, or predictive biomarkers available to reliably identify HGSOC patients who can benefit from PARPis therapy, and in particular, the subgroup with non-BRCA-mutant, HRD cancers [12, 24, 25, 31]. The routine identification of this class of HGSC tumors presents considerable challenges: the lack of evidence of a unifying pathological phenotype, the many components of the pathways and the numerous potential mechanisms of inactivation [32]. Moreover, taking under account the high cost of PARPis and NGS assays, the development of conceptually simpler and much less expensive predictive biomarker assays is essential.

As recently suggested, an alternative approach would be to develop functional assays that can detect HRD, regardless of the type of genetic aberrations that are present [25]. Previously, a functional assay for PARPis sensitivity in EOC was proposed [33], based on ascites-derived primary cell cultures (AsPCs) from EOC patients, grown as monolayers. The cytotoxicity to the PARPi rucaparib was tested in the AsPCs by survival assays, and the HR status was additionally investigated by γH2AX and RAD51 focus formation by immunofluorescence [33]. This pilot study was indicative of the possibility of using EOC cell-based assays for the analysis of PARPis sensitivity and HR function.

An alternative approach to drug testing is the use of 3D cell culture models (including spheroids and organoids), which better mimic primary tumors in vivo than traditional 2D cultures, due to the acquirement of additional tumor-like features like cell crowding and adhesion, hypoxia, nutrient deprivation, resistance to apoptosis, etc. [34–36]. Indeed, we and others have shown that 3D cultures display altered/reduced sensitivities to chemotherapeutic agents compared to 2D models, which may have a significant impact on the success of drug testing pipelines for cancer [36], including EOC [37–40].

Our primary objective was to develop a 3D functional assay to assess the sensitivity of two PARPis, niraparib and olaparib, in primary EOC cell cultures derived from ascites samples of HGSOC patients. We also compared the effectiveness of the functional assays when ascites-derived primary cultures (AsPCs) were grown both as 3D (spheroids) and monolayer cultures. Having established this 3D-based technique, we compared the cytotoxic effects of both PARPis in 42 AsPCs, and investigated if their HR status correlated with PARPis sensitivity. We also compared the gene expression patterns between sensitive and resistant AsPCs to both tested drugs to better understand the molecular mechanisms of the PARPis cytotoxic effect in AsPCs, and importantly, in an effort to identify biomarkers with putative predictive value to PARPis sensitivity in EOC patients.

## Materials and methods

### Patient cohort and ethical approval

Ovarian cancer patients included in this study were recruited in the period of September 2014 to September 2017 at the CHU de Quebec, Hôtel-Dieu Hospital in Quebec City, Canada. Ascites were collected from patients diagnosed with high-grade serous ovarian cancer (HGSOC), after obtaining written informed consent under protocols approved by the the CHU de Quebec Ethics Committee. The histopathological diagnosis, including tumor grade and stage were determined by pathologists as part of the clinical diagnosis. Ascites were obtained from patients immediately before primary surgery, or neoadjuvant chemotherapy. Most patients were treated with standard chemotherapy regimens (carboplatin + Taxol). All these patients developed recurrent disease within 6–20 months of first line of chemotherapy or surgery.

### Preparation of ascites-derived primary cell cultures (AsPCs)

A total number of 83 AsPCs were collected from patients; of the 83 AsPCs 42 AsPCs had the minimum volume required for proper cell culture and propagation. The volume of ascites varied among the individual HGSOC patients. In order to standardize the experimental protocol only a minimum volume of 50 ml of ascites was used to collect cells. Upon centrifugation for 5 min at 1200 rpm, the cell pellet was re-suspended in sterile MilliQ H_2_O, which removes the contaminating red blood cells due to hypotonic lysis. The bulk of ascites cells was then seeded on 1.5% agarose plates in OSE growth medium (representing 1:1 mix of Media 199 (Sigma-Aldrich, St. Louis, MS, USA) and MCDB 105 (Sigma-Aldrich, St. Louis, MS, USA), with the addition of 25 μg/mL gentamicin and 2.5 μg/ml fungizone, and supplemented with 10% fetal bovine serum. Cells were maintained at 37 °C in the presence of 5% CO_2_. Under these conditions, mostly epithelial tumor cells were able to form spheroids, which appeared floating over the agarose surface after a period of 2–3 days of incubation. Consecutively, floating spheroids were dispersed by pipetting and were counted for plating in 3D culture plates for further experimentation. In parallel, floating spheroids were similarly dispersed by pipetting and tumor cells were plated and maintained as monolayers in plastic tissue culture flasks supplemented with OSE growth medium, as described above. The AsPCs thus obtained were passaged weekly and experiments were performed within 2–3 passages.

### AsPCs spheroid formation in hanging drops using 96-well plates

We used the Perfecta3D 96-Well Hanging Drop Plates (Sigma-Aldrich, St. Louis, MS, USA) for spheroid formation in hanging drops. These plates are currently discontinued and replaced by similar plates of the Nunclon Sphera 3D culture system (Thermo Fisher Scientific, Inc., Waltham, MA, USA). The protocol for proper cell plating using the system consisted of the addition of 2 mL of water to the reservoirs located on the peripheral rim of the plate and tray. Five thousand cells in 50 μL of media volume were plated per well. Hanging drops were thus formed and confined to the bottom of the plate. Within hours of plating, individual cells initiated to aggregate and eventually formed into spheroids. Spheroid formation time varied between patients ranging between 2 and 4 days. Cell culture media was replaced every day or every other day. The media exchange protocol consisted of removing 10 μL media without disturbing the spheroid and replacing it with 15 μL of fresh media.

### Olaparib and niraparib treatment

AsPCs spheroids in hanging drops or AsPCs plated as monolayers on plastic plates were exposed to increasing concentrations of either olaparib (AZD-2281)-(AstraZeneca, Wilmington, DE, USA) or niraparib (MK4827)-(Merck, Kenilworth, NJ, USA) at the following concentrations (0 μM, 10 μM, 50 μM and 100 μM). In some experimental conditions, cells were additionally treated with 100 μM etoposide. The AsPCs were incubated with the drugs for 72 h and survival or toxicity was determined using either the WST-1 assay for the hanging drop (spheroid) model or applying the MTS assay for the cells growing in monolayer.

### WST-1 cell viability assay for hanging drops AsPCs (spheroids) cultures

For AsPCs growing in the hanging drops, the Perfecta3D cell viability kit (Sigma-Aldrich, St. Louis, MS, USA) was used following the user’s manual. The Perfecta3D cell viability kit has been discontinued, the material supplied in the kit including the WST-1 reagent, the mediator solution, and the round bottom clear plates can be purchased seperatly from Abcam (Cambridge UK). Briefly, at the end of the treatment period, water was first removed from the side reservoirs of the 3D cell culture plates. Then, the bottom of the tray was removed from the 3D cell culture plate, and the top plate with the hanging drops was placed on top of a round bottom clear plate making sure that the wells of the top and bottom plates are lined up. The hanging drops were transferred to the wells of the bottom plate by spinning the plates at 200× *g* for 1 min at room temperature. The upper (3D culture) plate was then removed from the round bottom plate and 5 µl of the WST-1 solution was added to each well. The solution was mixed gently for one minute on an orbital shaker and the spheroids were incubated at 37 °C incubator for 4 h. After the 4 h incubation, the plate was mixed gently on an orbital shaker for one minute to ensure homogenous distribution of color. The absorbance was measured using a microplate reader at a wavelength of 450 nm. Each experiment was performed in triplicates, and the mean value was calculated. The percentage of cell viability was normalized to the dimethylsulphoxide (DMSO) control.

### MTS cell viability assay for monolayer cultures

For AsPCs growing in monolayer, cell viability was assessed using the MTS colorimetric assay (Promega, Madison, WI, USA) following the user’s manual. AsPcs were seeded in 96-well plates in 100 μl complete medium at a density of 15 × 10^4^ cells per well and incubated with olaparib or niraparib, and olaparib or niraparib in combination with etoposide. Twenty μl of the MTS reagent (Promega, Madison, WI, USA) was added to each well and the plates were further incubated for additional 2 h. The absorbance was measured using a microplate reader at a wavelength of 450 nm. Each experiment was performed in triplicates, and the mean value was calculated. The percentage of cell viability was normalized to the dimethylsulphoxide (DMSO) control.

### Cell viability statistical analysis

The concentration of the drug required to reduce cell viability by 50% at 72 h treatment (i.e. the IC50 of olaparib or niraparib) was initially determined. The IC50 values of olaparib or niraparib, were used to evaluate the sensitizing effect of each drug. Comparisons between the treatments were performed using repeated measures analysis of variance (ANOVA). Whereas differences between means were inspected with Dunnett’s multiple comparison post hoc tests. A *p* value < 0.05 was considered statistically significant. Cell viability assays were represented as mean ± S.D. All expxeriments were performed in triplicates. The data was analyzed using the Prism software.

### Immunoflourescence

AsPCs displaying either sensitivity or resistance to either drug treatment (olaparib and/or niraparib) were plated again on 1.5% agarose plates and allowed to form spheroids, which were then dispersed and grown in monolayer on poly-L-lysine (Sigma-Aldrich, St. Louis, MS, USA) coated coverslips for 48 h. Cells were washed once with PBS, then incubated with gentle agitation for 5 min at room temperature in permeabilization buffer consisting of 0.5% Triton™ X-100 (Sigma-Aldrich) in PBS. Cells were then incubated with gentle agitation for 1 h at room temperature in blocking buffer consisting of BSA (Sigma-Aldrich, St. Louis, MS, USA) with 0.1% Triton™ X-100 in PBS. The blocking buffer was removed and the cells were further incubated with gentle agitation overnight at 4 °C in primary antibody solution: (N-cadherin (1:250) (Abcam Branford, CT, USA), E-cadherin (1:250) (Abcam Branford, CT, USA), and anti-phospho-histone H2AX (serine 139), mouse monoclonal IgG1 antibody, clone JBW301 (1:500) (Millipore, Burlington, MA, USA), all diluted in blocking buffer. Excess primary antibody was removed by washing twice with washing buffer, consisting of 0.1% Tri- ton™ X-100 in PBS. Subsequently slides were incubated with secondary antibodies, including rhodamine-linked goat-anti-mouse IgG1 (Santa Cruz Biotechnology Dallas, TX, USA) or Alexa Fluor 488-labeled goat anti-rabbit antibody (Abcam, Branford, CT, USA) diluted 1:1000 in blocking buffer. Cells were finally stained with 4′,6-diamidino-2-phenylindole (DAPI). Refer to Additional file 1 for details on the antibodies used. Images were captured using a Zeiss LSM 700 confocal microscope (Carl Zeiss Meditec AG Jena, Germany). yH2AX puncta were counted per cell on a minimum number of 30 cells per condition. All experiments were performed in triplicates. The one-way Anova-Tukey's multiple comparisons test was used for statistical analysis. A significant association was considered when *p*-values were < 0.05. All values were expressed as the means ± S.D. The data was analyzed using the Prism software.

### Western blot analysis

Whole-cell lysates were prepared from the AsPCs cell pellets using the RIPA lysis buffer. Equal amounts of protein extracts were resolved by SDS-PAGE and were transferred to PVDF membrane (Bio-Rad, Hercules, CA, United States). The primary antibodies (N-cadherin (1:1000) (Santa-Cruz, Dallas, TX, USA), E-cadherin (1:1000) (Santa-Cruz, Dallas, TX, USA), and (1:1000) beta-actin (Santa-Cruz, Dallas, TX, USA) were used; refer to Additional file 1 for details on the antibodies used. The blots were developed using the chemiluminescent detection system (ECL) (Santa-Cruz, Dallas, TX, USA). All Western blots were performed in at least three independent experiments. The comparison of protein expression levels was performed using Student's t test. A significant association was considered when *p*-values were < 0.05. All values were expressed as the means ± S.D. The data was analyzed using the Prism software.

### Gene expression profiling and data analysis

Gene expression analysis was carried out as previously described [41]. Total RNA was extracted from all 3D cultured AsPCs used in the study using RNeasy Plus Mini Kit (Qiagen, Hilden, Germany). The quality of the RNA samples was examined by capillary electrophoresis using the Agilent 2100 Bioanalyzer (Agilent, Santa Clara, CA, USA). Fluorescently labeled cRNA targets were generated from 0.5 μg of total RNA from each of the AsPCs, using the Fluorescent Linear Amplification Kit (Agilent, Santa Clara, CA, USA) and 10 mM Cyanine 3- or 5-labeled CTP (PerkinElmer, Waltham, MA, USA), following the user’s manual. Cyanine-labeled cRNAs from randomly paired 6 olaparib- and niraparib sensitive and 6 olaparib- and niraparib-resistant AsPCs were mixed with the same amount of reverse-color cyanine-labeled cRNA from their corresponding counterpart (Sensitive vs. Resistant) and hybridized on the Agilent Whole 4 × 44 K Human Genome microarrays. All microarray experiments were performed in duplicates using a dye reversal (dye-swap) labeling technique. Pathway and network analyses were completed using the IPA software (see https://www.Ingenuity.com). The microarray data have been deposited to the GEO database (https://www.ncbi.nlm.nih.gov/geo/) with accession number GSE149940.

### Quantitative PCR (qPCR)

For RT-qPCR, first total RNA was extracted using the RNeasy Plus Mini Kit (Qiagen, Hilden, Germany). RNA was then reverse-transcribed into cDNA using Superscript III transcriptase, according to the manufacturer’s protocol (Invitrogen; Thermo Fisher Scientific, Inc., Waltham, MA, USA). RT-qPCR was performed using the SYBR Green PCR Master Mix (Applied Biosystems; Thermo Fisher Scientific, Inc., Waltham, MA, USA) on a ROTOR GENE real-time PCR machine (Corbett Robotics, Qiagen, Hilden, Germany). Primers were designed as previously shown [42]; with the sequences freely available from the Entrez Nucleotide database and the Primer3 algorithm for primer design (https://www-genome.wi.mit.edu/cgi-bin/primer/primer3_www.cgi). All primers for qPCR are listed in Additional file 2. PCR volume was 20 μl, and conditions were as follow: initial cycle 50 °C, 2 min, 95 °C, 15 min; 45 cycles at 95 °C, 20 s, 60 °C, 20 s and 72 °C, 20 s; final cycle 72 °C, 30 s. Data were analyzed by the Rotor-Gene software using the comparative ΔΔCt method. The relative copy number was calculated based on the target gene/18S RNA ratio.

All values were expressed as the means ± S.D. Each sample was tested in triplicate.

### Tissue micro arrays (TMAs) and immunohistochemistry (IHC)

TMAs were constructed as previously described [43]. Briefly, one representative block of each ovarian tumor used for validation was selected for the preparation of the tissue arrays. Three 0.6-mm cores of tumor were taken from each tumor block and placed, 0.4 mm apart, on a recipient paraffin block using a commercial tissue arrayer (MTA-II arrayer) (Beecher Instruments, Hummingbird, WI USA). The cores were randomly placed on the recipient blocks to avoid evaluation biases.

IHC analyses were performed on 4-μm tissue sections, which were deparaffinized and rehydrated in graded alcohols, then incubated with blocking serum for 20 min. Following treatment with 3% H_2_O_2_ for 10 min to quench the endogenous peroxidise activity, sections were incubated with the primary antibody overnight (C-Met 1:150 (LSBio, Seattle, WA, USA, monoclonal, LS-C96426), CDKN2A 1:100 (Abcam, Cambridge, UK, monoclonal, ab54210), P-glyc (ABCB1) 1:100 (Abcam, Cambridge, UK, monoclonal, ab168337), FANCF 1:100 (LSBio, Seattle, WA, USA, polyclonal, LS‐B210), SPRY2 1:75 (Abcam, Cambridge, UK, monoclonal, ab60719), E-cadherin 1:100 (Abcam, Cambridge, UK, polyclonal, ab15148), and N-cadherin 1:100 (Abcam, Cambridge, UK, polyclonal, ab76057) at 4 °C. Refer to Additional file 1 for details on the antibodies used. Incubation and detection with SignalStain 3,3′-diaminoben-zidine (DAB) Substrate kit (IDetect Universal Mouse Kit HRP-DAB; ID Labs, Pittsburgh, PA, USA) were done according to the manufacturer's instructions. Sections were then counter-stained with hematoxylin. Images were acquired using a Leica Confocal Scope (TCS SP5 X; Leica Microsystems, Wetzlar, Germany) and analyzed via the Leica Application Suite Software (Leica Microsystems, Wetzlar, Germany).

### TMA scoring and statistical analysis

Protein expression was scored according to intensity (value of 0 for absence, 1 for low, 2 for moderate, and 3 for high) of staining based on manual visualization. A composite score was defined as the product of staining intensity (nuclear, cytoplasmic, or membranous depending on the expected staining). All slides were independently scored in a blinded manner by 2 observers, and the integration was > 85%. In case of differences between the 2 scorings, the core was re-evaluated to reach a consensus. The relationship between the protein expression of the listed genes was evaluated by the Wilcoxon two-sample test. A significant association was considered when *p*-values were < 0.05. All values were expressed as the means ± S.D. The data was analyzed using the Prism software.

## Results

### Establishing ascites-derived monolayer and 3D (spheroid) primary cell cultures

**Fig. 1:**
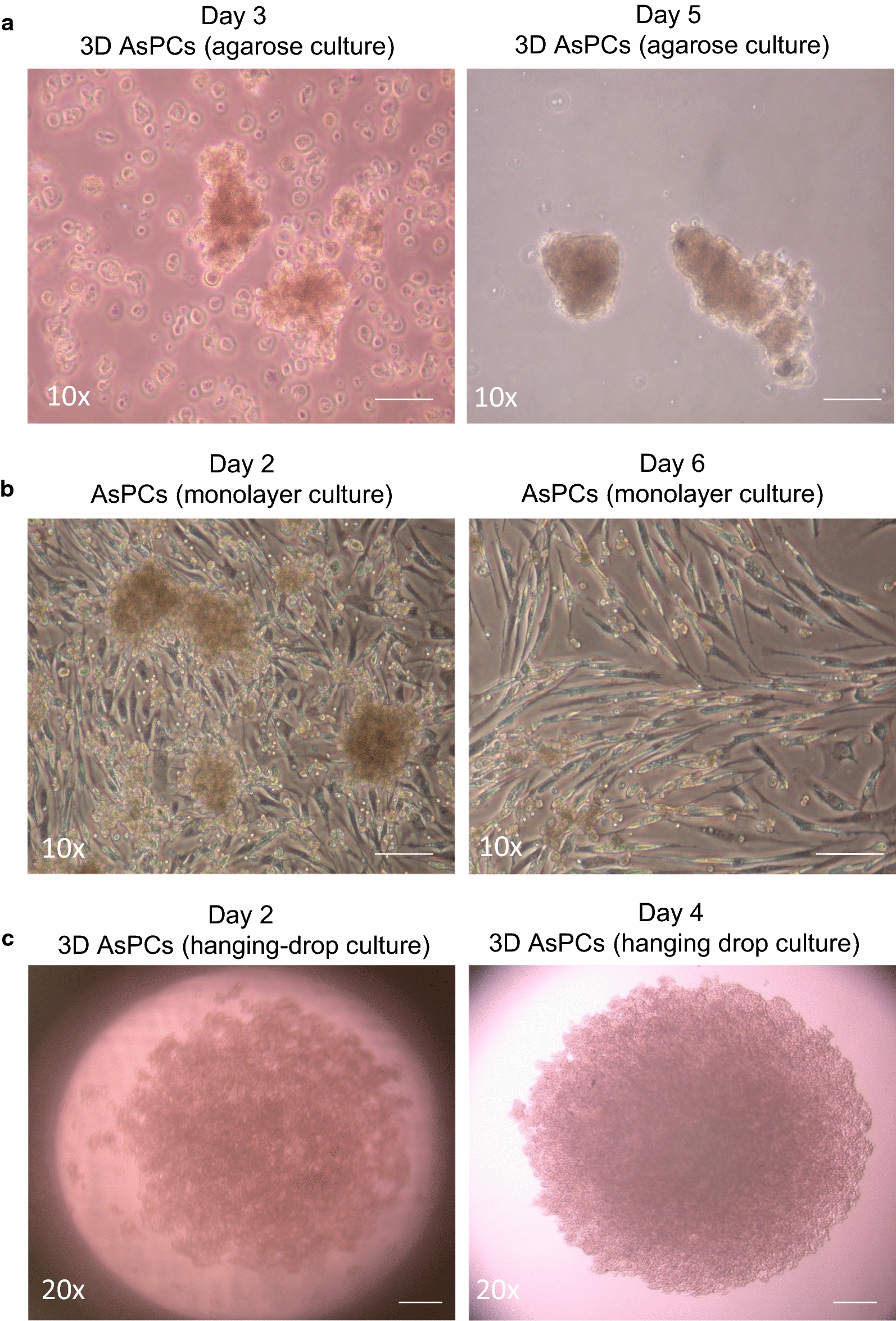
Establishing ascites-derived monolayer and 3D (spheroid) primary cell cultures. **a** Spheroid AsPCs seeded on agarose plates 3 days and 5 days post collection. Scale bar = 50 mm. **b** Morphological features of spheroid AsPCs grown on tissue culture plastic plates 2 days and 6 days following seeding from agarose plates to monolayer. Scale bar = 50 mm. **c** Morphological features of AsPCs spheroids grown in 3D hanging drops 2 days and 4 days post seeding from agarose plates. Scale bar = 20 mm. The images are representative of (n = 25) samples

We developed a simple method for ascites-derived primary cell cultures (AsPCs) with almost 100% success rate of AsPCs propagation, when using at least 50 ml startup volume of ascites sample. AsPCs derived from HGSOC patients were grown in culture for 3 to 5 days on 1.5% agarose plates, allowing ascites multicellular aggregates (MCAs) to quickly grow and acquire a spheroid morphology (Fig. 1a). Using this approach, we were able to eliminate cells growing as monolayers (Fig. 1a). Upon gentle dispersion of the spheroid culture, cell homogenates (containing spheroid-like aggregates) were grown as monolayers (Fig. 1b) or 3D (spheroid) cultures in hanging drop plates (Fig. 1c); see Materials and Methods for details. Cell homogenates, when grown in monolayer, went through a clear transformation, as the spheroid-like cell aggregates gradually dispersed into adherent monolayer cultures, which after 6 days in culture developed elongated, mesenchymal-like morphology (Fig. 1b). Cell homogenates transferred to the 3D hanging drop plates maintained their structural morphology, presenting with one compact spheroid structure around day 4 post-plating (Fig. 1c). AsPCs grown as monolayers varied in their growth potential: the first passage was carried out between 5 and 7 days following collection. Senescence occurred between the third and eighth passages, most commonly between fourth and sixth.

### Cytotoxicity assays in 3D and monolayer AsPCs upon treatment with olaparib and niraparib

We performed cytotoxicity assays for AsPCs, both grown in 3D (spheroid) and as monolayer cultures, to better understand which cellular model better mimics patients’ response to PARPis-targeted therapy. AsPCs, grown as spheroids and in monolayer, were treated with olaparib and niraparib—two of the FDA-approved PARPi drugs used for EOC management [11]. We used the Cayman’s Perfecta 3D Cytotoxicity Assay for analysis of PARPis cytotoxicity of AsPCs grown as spheroids, which allows for direct assessment of drug’s cytotoxicity in a single hanging drop spheroid culture. Using this approach, cells were grown in triplicates, and were treated with increasing concentrations of the PARPis olaparib and niraparib (alone, or in combination with 100 μM of etoposide) over a period of 72 h. Likewise, AsPCs grown in monolayer were treated in parallel over a period of 72 h with either olaparib or niraparib (alone, or in combination with 100 μM of etoposide). When identifying AsPCs as either PARPi resistant or sensitive, treatment response was based on toxicity effects of niraparib or olaparib alone. Thus, when examining treatment response at the monolayer level, we found that from a total of 42 AsPCs used in this study, 37 AsPCs (88%) displayed strong sensitivity to niraparib treatment, and only 5 AsPCs (12%) displayed a clear resistance to niraparib treatment. Accordingly, 22 AsPCs (52%) displayed high sensitivity to olaparib, and 20 AsPCs (48%) were olaparib-resistant (see Additional file 3A). Thus, AsPCs grown in monolayer, showed to have significantly higher sensitivity (36%) to niraparib treatment, as compared to that of olaparib.

**Fig. 2:**
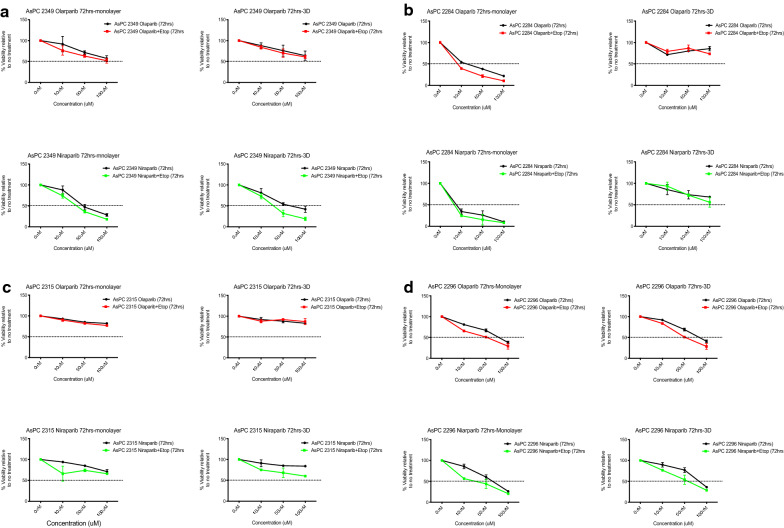
Cytotoxicity assays in 3D and monolayer AsPCs upon treatment with olaparib and niraparib. Cytotoxicity of monolayer (left) and 3D (right) using olaparib top and niraparib bottom of: **a** olaparib resistant (both in monolayer and 3D) and niraparib sensitive (both in monolayer and 3D) AsPCs; **b** olaparib and niraparib sensitive in monolayer, and resistant in 3D AsPCs. **c** olaparib resistant and niraparib resistant AsPCs in monolayer and 3D; **d** olaparib sensitive and niraparib sensitive AsPCs in monolayer and 3D. Toxicity response was determined using two independent assays (MTT for monolayer, and WTS for 3D). Cells were treated with either olaparib (0–100 μM) or niraparib (0–100 μM) and in combination with etoposide (100 μM) 24 h after cells were seeded. Toxicity assays were performed 3 days after treatment. The cell viability was calculated relative to the 0.01% DMSO-treated control AsPCs. One representative cell viability plots from 2 independent experiments are shown. All values were expressed as the means ± S.D of the 3 replicates used in the toxicity assay

Similarly, when examining treatment response at the 3D level, 28 AsPCs (66%) were sensitive to niraparib, and 14 (34%) AsPCs were resistant to niraparib, while 16 AsPCs (38%) displayed sensitivity to olaparib, and 26 AsPCs (62%) revealed to be resistant to olaparib (see Additional file 3B). Likewise, treatment response to niraparib was significantly higher (28%) in 3D AsPCs, as compared to that of olaparib. Thus, for the majority of AsPCs included in this study, the cytotoxic effect of niraparib was considerably stronger in AsPCs grown in monolayer and 3D, as compared to that of olaparib (see Additional file 3). A representative example of such toxicity trend is presented with AsPC 2349 (Fig. 2a).

Cytotoxicity data from both approaches (monolayer and 3D) confirmed that significantly larger number of 3D AsPCs displayed drug resistance as compared to their monolayer counterparts, similar to the observations made with the example AsPC 2284 (Fig. 2b). Nonetheless, several AsPCs did demonstrate a comparable trend in sensitivity and resistance upon niraparib and olaparib treatment. Moreover, cytotoxcity assays indicated that 16 (38%) AsPCs were sensitive to olaparib and niraparib in both monolayer and 3D culture, while 6 (14%) AsPCs were resistant to olaparib and niraparib in both monolayer and 3D culture, as shown for AsPCs 2315 and 2296 respectively (Fig. 2c and d).

### Analysis of the HR pathway activity (γH2AX foci formation) in PARPis-sensitive and resistant AsPCs

**Fig. 3:**
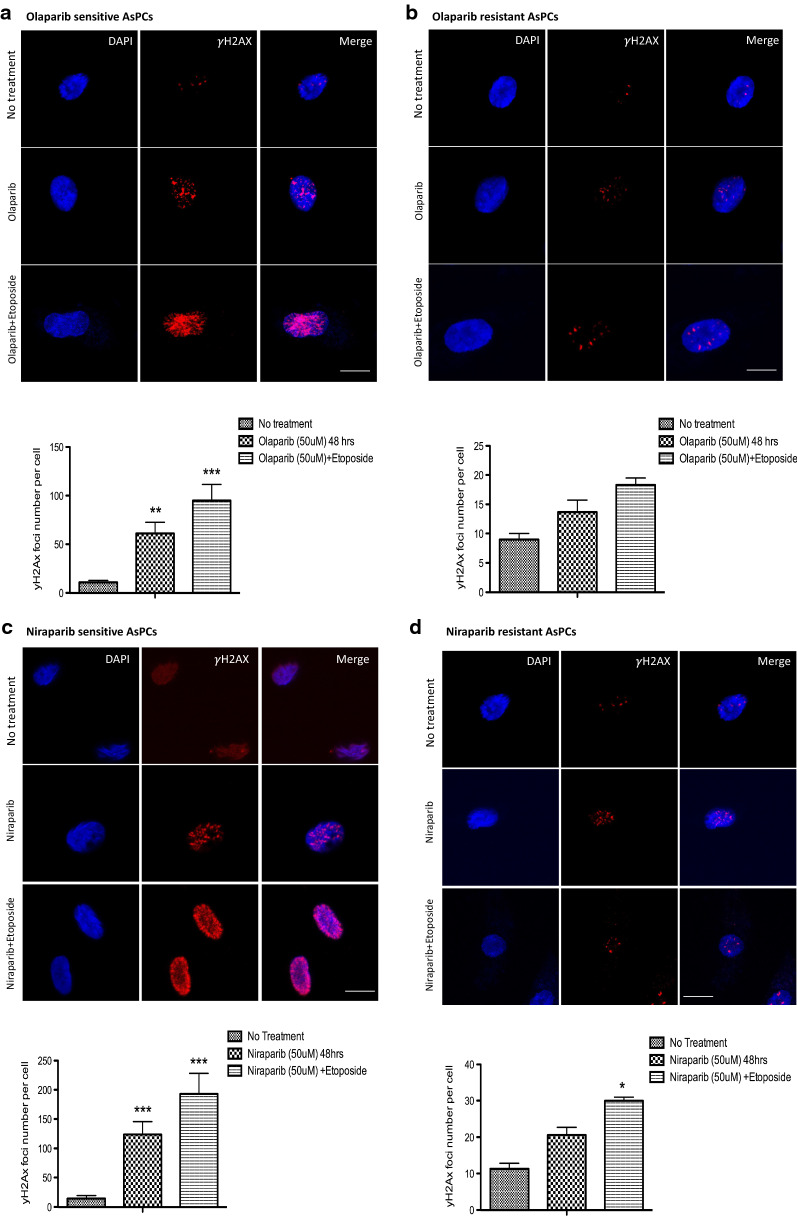
Analysis of the HR pathway activity (γH2AX foci formation) in PARPis-sensitive and resistant AsPCs. Comparative analysis of olaparib and niraparib induced formation of γ-H2AX foci. **a** Representative images of olaparib-induced foci formation in olaparib-sensitive AsPCs vs. **b** olaparib-resistant AsPCs. AsPCs were exposed to olaparib alone or to olaparib with etoposide for 48 h, the γ-H2AX foci formation was detected by immunofluorescence. **c** Niraparib-induced foci formation in niraparib-sensitive AsPCs vs. **d** niraparib-resistant AsPCs. AsPCs were exposed to niraparib alone or niraparib with etoposide for 48 h, the γ-H2AX foci formation was detected by immunofluorescence. Representative histograms are shown with DMSO used as the no treatment control (n = 3). The multiple comparison-one-way Anova-Tukey's multiple comparisons test was used for statistical analysis. Error bars denote standard deviation of each mean calculation. *p < 0.05 **p < 0.01 and ***p < 0.001. Scale bar = 10 µm

Since PARP inhibition results in reduced DNA repair mechanisms, we further validated our results by examining the HR status in selected PARPis-sensitive and resistant AsPCs (at the 3D level) using the γH2AX foci formation assay. The assessment of γH2AX foci formation was performed by immunofluorescence following treatment with either olaparib or niraparib alone, or in combination with etoposide. Upon 48 h treatment, we found that γH2AX foci formation was significantly higher in niraparib-sensitive AsPCs compared to olaparib-sensitive AsPCs, and this effect was especially stronger when AsPCs were treated in combination with the DSBs inducer etoposide (Fig. 3a and c), suggesting that olaparib is a weaker inducer of the DNA damage marker γH2AX in the PARPis-sensitive AsPCs. Furthermore, AsPCs that were resistant to either niraparib or olaparib alone or in combination with etoposide, displayed no significant differences in γH2AX foci formation as compared to the non-treated condition (Fig. 3b and d). Thus, the functional analysis of HR status confirmed our observations for the stronger cytotoxic effect of niraparib in both monolayer and 3D AsPCs, when compared to that of olaparib.

### PARPis sensitive and resistant AsPCs present with different EMT features

Since epithelial to mesenchymal transition (EMT) has been strongly involved in mechanisms of resistance to EOC therapy [44], including PARPis resistance [45], we examined the expression of the two major EMT markers, E-cadherin and N-cadherin, in a panel of PARPis-resistant and PARPis-sensitive AsPCs (based on their response to treatment in 3D (spheroid) cultures). Both Western blot (see Additional file 4A) and immunofluorescence (see Additional file 4B) analyses were indicative of significantly higher N-cadherin protein expression levels in PARPis-resistant AsPCs compared to their sensitive counterparts, while no significant differences were observed when examining E-cadherin protein expression levels in both sensitive and resistant AsPCs.

**Fig. 4:**
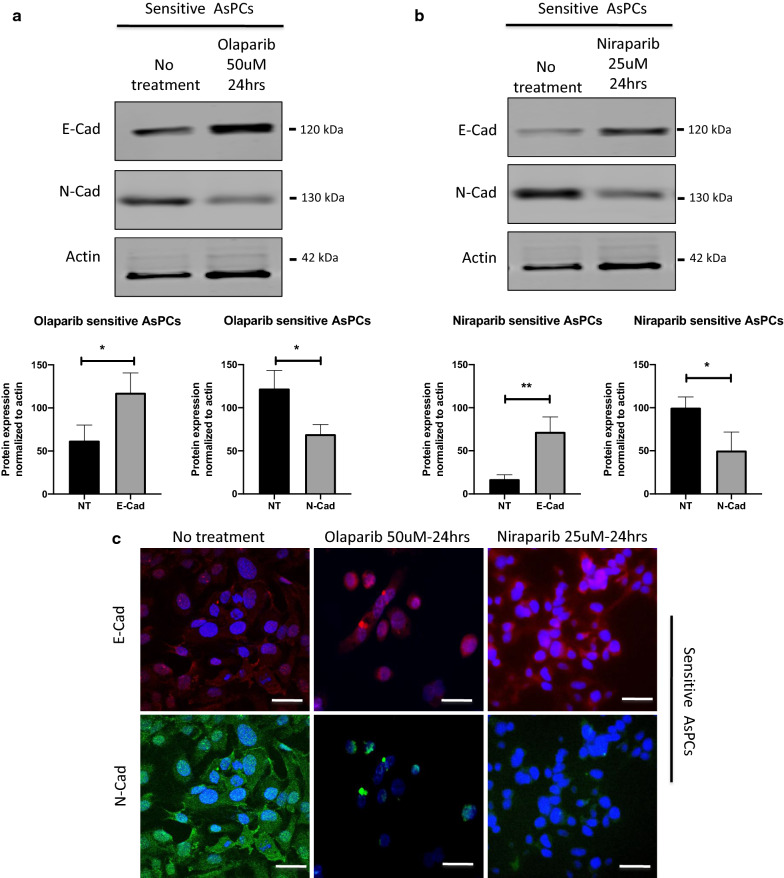
The effect of olaparib and niraparib on EMT in PARPis-sensitive AsPCs. AsPCs were grown in monolayers for 48 h and then treated with either olaparib at a concentration of 50 μM for a period of 24 h, or niraparib at a concentration of 25 μM for a period of 24 h, as non-treated AsPCs were used as controls. Western blot protein expression analysis of the two EMT markers, E-cadherin and N-cadherin in **a** olaparib-sensitive AsPCs and **b** niraparib-sensitive AsPCs. Actin was used as the loading control (n = 3). Histograms represent 3 PARPis-sensitive AsPCs, and protein expression levels were normalized to actin. The two-tailed unpaired t-test was used for statistical analysis. All values were expressed as the means ± S.D. *p < 0.05 **p < 0.01 and ***p < 0.001 **c** Immunofluorescence analysis of the two EMT markers E-cadherin and N-cadherin in sensitive AsPCs as compared to the no treatment control. Scale bar = 20 µm

Interestingly, olaparib and niraparib treatment of 3D sensitive AsPCs showed a clear and significant increase in E-cadherin protein expression levels and a significant decrease in N-cadherin protein expression levels in several of the AsPCs studied, as examined by Western blot (Fig. 4a and b) and confirmed by immunofluorescence analyses (Fig. 4c). In contrast, both PARPis treatment had no effect on the expression of both these EMT markers in resistant 3D AsPCs (see Additional file 5).

### Comparative analysis of the molecular mechanisms of PARPis action in PARPis-sensitive and resistant AsPCs

To better understand the molecular mechanisms of PARPis cytotoxic effect in AsPCs, we employed the Agilent whole human genome 4 × 44 K microarrays (containing 44,000 genes) to identify gene expression alterations between PARPis-sensitive and resistant AsPCs. AsPCs grown in 3D culture, which displayed sensitivity or resistance to both PARPis, were selected for microarray analysis. Thus, 6 sensitive and 6 resistant AsPCs (randomly paired) were compared, as the microarray experiments were performed in duplicates using a dye reversal (dye-swap) labeling technique. For all comparisons, a subset of differentially expressed genes were selected by initial filtering on confidence at *p*-value ≤ 0.05, followed by filtering of expression level (≥ 1.5 fold). Using these selection criteria, we found 240 upregulated genes and 583 downregulated genes in the PARPis-sensitive AsPCs, as compared to the PARPis-resistant AsPCs (see Additional file 6).

Consecutive network analyses generated through the Ingenuity Pathway Analysis (IPA) software were indicative of major gene nodes linked to important pathways related to EOC tumorigenesis. Interestingly, numerous gene nodes and networks with previously demonstrated functional implications in EOC chemoresistance (including calpain, P-glycoprotein/*ABCB1*, *PBX1, LGALS8, CARD10, GST, LAMB1, AHR, IFI16, ATPase*, the *MAPK* and *MEK* networks) were found to be down-regulated in the 6 PARPis-sensitive AsPCs, when compared to the 6 PARPis-resistant AsPCs, and only a few gene nodes associated with EOC chemoresistance *(DAXX, RAS and Vim*) displayed up-regulation in PARPis-sensitive AsPCs (see Additional file 7). A number of gene nodes linked to drug resistance in other cancer types (including *CAT, TRIM59, ANXA2, ADM, AJUBA, HSPA1A/1B, LAMC2, TRIM14, KRT8, KRT18, KRT9, CLDN1,* and *SMARCA2*) were also down-regulated in PARPis-sensitive AsPCs (see Additional file 7).

Moreover, common IPA canonical pathway analyses were indicative of major oncogene-related signaling pathways that were differentially modulated between PARPis-sensitive and resistant AsPCs. Thus, the most significantly downregulated canonical pathways in PARPis-sensitive AsPCs, as compared to the PARPis-resistant AsPCs, were related to antigen presentation pathways, interferon signaling, toll-like receptor, TGF-β, IL-6 and p38 MAPK signaling (Additional file 8A). Accordingly, upregulated canonical pathways in PARPis-sensitive AsPCs, when compared to PARPis- resistant AsPCs, were predominantly associated with the Rho family GTPases, JNK, NGF and PDGF signaling (Additional file 8B).

### Identification of potential biomarkers, differentially expressed in PARPis- sensitive and resistant AsPCs and their matched HGSOC tumors

For further validation of the microarray data, the expression levels of a panel of 24 differentially expressed genes were analyzed in the 6 PARPis-sensitive and 6 PARPis-resistant AsPCs included in our microarray experiments (validation set), as well as in an additional set of 6 PARPis- resistant and 6 PARPis- sensitive AsPCs (test set). The 24 genes chosen for validation analysis (listed in Additional file 9) were selected based on their previously described role in tumorigenesis (including EOC tumorigenesis), implications in DNA damage response and HR function, and EMT regulation. The expression levels of the 24 selected genes were examined by quantitative PCR (qPCR) in both the validation and the test sets, and were compared with their microarray-based expression values (microarray). As shown in Additional file 9, the qPCR analysis confirmed the validity of our microarray data readout, as the expression of the 24 genes was highly concordant in all three experimental sets analyzed.

**Fig. 5:**
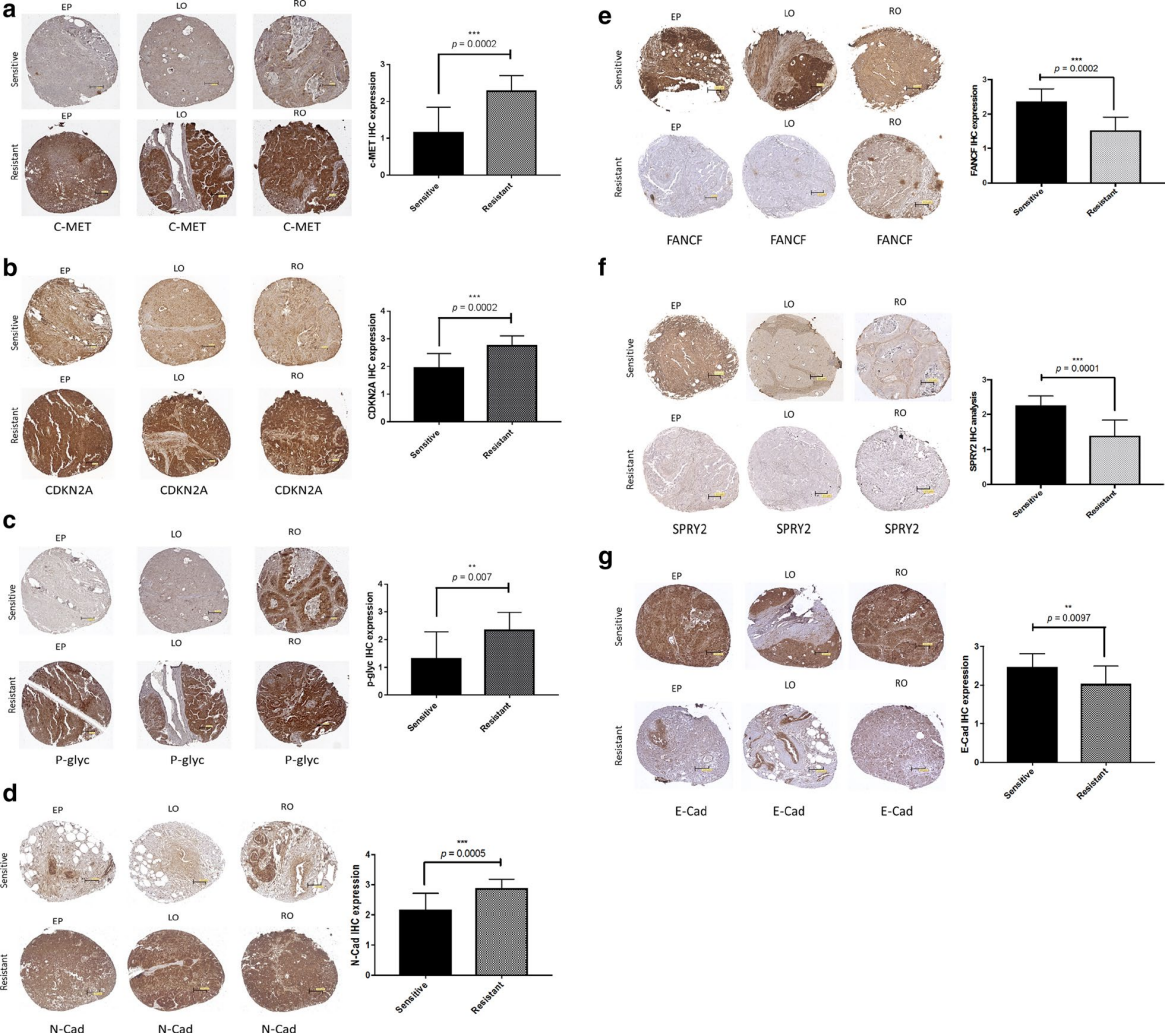
Identification of potential biomarkers, differentially expressed in PARPis- sensitive and resistant AsPCs and their matched HGSOC tumors. Protein expression analysis of the seven genes c-Met, CDKN2A, P-glyc, N-cadherin, FANCF, SPRY2, and E-cadherin in HGSOC tumor samples, matched to PARPis-sensitive and resistant AsPCs. **a–d** c-Met, CDKN2A, P-glyc and N-cadherin staining patterns in representative cores in epiploon (EP), left ovary (LO) and right ovary (RO) tumors, comparing sensitive (top) vs. resistant (bottom) tissue samples. **e–g** FANCF, SPRY2 and E-cadherin staining patterns in representative cores in epiploon (EP), left ovary (LO) and right ovary (RO) tumors, comparing sensitive (top) vs. resistant (bottom) tissue samples. Box-plot representation of the protein expression levels in sensitive vs. resistant ovarian tissues are presented next to each of the TMAs. The Wilcoxon two-sample test was used for statistical analysis. All values were expressed as the means ± S.D. *p < 0.05 **p < 0.01 and ***p < 0.001

In search of potential biomarkers for response to the PARPis studied, we further analyzed the protein expression levels of 5 genes of the 24 initially selected genes by IHC using TMAs containing matched tumor samples derived from the corresponding 12 sensitive and 12 resistant AsPCs included in our validation and test sets. Moreover, we also examined the protein expression levels of the two EMT markers: E-cadherin and N-cadherin. The seven analyzed potential biomarkers exhibited similar expression in the matched tumor samples, corresponding to their expression patterns in the PARPis-sensitive and resistant AsPCs. Thus, C-MET, CDKN2A, P-glyc (ABCB1) and N-cadherin displayed significantly lower protein expression levels in HGSOC tumors corresponding to PARPis-sensitive AsPCs (Fig. 5a–d), while FANCF and SPRY2 showed stronger expression in the PARPis-sensitive AsPCs-matched tumor samples (Fig. 5e–f). Additionally, E-cadherin also showed significantly higher expression levels in PARPis-sensitive AsPCs-matched tumor samples (Fig. 5g).

## Discussion

Research in oncology is now becoming more aware of the importance of 3D culture applications to understand how tumors develop, including their utility in compound screening for predicting drug response in patients [46, 47]. Studying prolonged and long term effects of treatments is more feasible in a 3D setting due to the lower proliferation rates exhibited by cells in 3D cultures [48], in addition to lower levels of apoptosis, as well as various parameters relating to cellular motility, and cell morphology [49]. Likewise, studies have also shown that not only treatment response profiles are different between monolayer and 3D cultures, but also their gene expression profiles appear to be very different. There is accumulating evidence that 3D models display much closer similarities to in vivo gene expression profiles, including genes involved in cellular adhesion, proliferation, immune response and cellular organization pathways [50–53].

Different 3D cell culture models have been applied to study EOC dissemination and response to therapy (recently reviewed in [39, 54, 55]), including the application of microfluidic technologies based on 3D spheroid-based sampling [54, 56]. Since the fallopian tubes have been recently recognized as a potential primary origin of HGSOC [57], organoid cultures have been also produced from human and mouse fallopian tube epithelium [58–60]. However, some concerns have been raised about using the organoid models in cancer research, as the large number of growth factors added to the organoid media and the variable time required for their generation may lead to epigenetic changes and in vitro selection of specific tumor clones [61–64].

The main objective of our study was to develop a 3D functional assay based on AsPCs derived from HGSOC patients, for assessing the treatment response to two FDA-approved PARPi drugs (olaparib and niraparib). We initially established a method for AsPC preparation based on a matrix (agarose) which allows for easy isolation and successive propagation of AsPCs, grown as monolayer or as 3D cultures. The method can be also successfully applied for PCs preparation from solid tumors, upon mechanical disruption and consecutive collagenase treatment of the tumor tissue (data not published). As seen from Fig. 1b, monolayer AsPCs obtained by this method displayed a mesenchymal (spindle-like) cellular morphology after 6 days in culture; a phenomenon, frequently described by others [33, 65]. These cell cultures were successfully used for the preparation of our 3D (hanging drop) AsPC model. Indeed, it was previously shown that mesenchymal-type EOC cells can easily aggregate into compact solid spheroids, as compared to epithelial-type EOC cells, which rather form loose and unstable MCAs [66, 67]. Based on this method, we have developed a 3D AsPCs functional assay, which is relatively simple and can be carried out in a time frame compatible with its use as a tool to direct subsequent therapy. Moreover, we performed cytotoxicity assays on AsPCs grown both as monolayers and spheroids in order to compare their effectiveness and more importantly, to highlight the advantage of the 3D cellular model in predicting response to PARPis treatment. As seen from our data, the response to PARPis treatment in AsPCs, when grown as monolayers, was significantly higher, compared to that in spheroid AsPCs, as 88% and 52% of the monolayer AsPCs displayed sensitivity to niraparib and olaparib respectively. Accordingly, 66% AsPCs were sensitive to niraparib and 38% to olaparib at the 3D level. Although it might be premature to extrapolate these data obtained from 42 AsPCs, the sensitivity responses obtained by both PARPis at the 3D level are more or less consistent with previous estimates of HRD (40%–60%) in EOC [19, 68, 69]. Moreover, in both cellular model systems (monolayer and 3D), niraparib displayed a significantly stronger cytotoxic effect in AsPCs, which was further confirmed by our consecutive analyses of the HR pathway activity (γH2AX foci formation) in PARPis-sensitive and resistant AsPCs. These findings show that there is a very good correlation between HR status of the AsPCs and their sensitivity to PARP inhibition, essentially pointing to a possible consideration of niraparib as a PARPis-preferable therapeutic option in treating of EOC patients.

Our data are also indicative for significantly higher expression levels of the mesenchymal EMT marker N-cadherin in resistant 3D AsPCs when compared to their sensitive counterparts. Indeed, acquisition of the mesenchymal phenotype in EOC has been frequently shown to be particularly associated with aggressive metastatic invasion and chemoresistance [70–72]. Importantly, treatment of sensitive 3D AsPCs with both PARPis resulted in N-cadherin suppression and significant induction of E-cadherin expression, while PARP inhibition had no effect on the expression of both these EMT markers in resistant 3D AsPCs.

Further, microarray experiments focused on comparing the gene expression patterns between 6 sensitive and 6 resistant AsPCs to both tested drugs, and consecutive IPA network and pathway analyses, were indicative for the predominant downregulation of numerous genes and networks with previously demonstrated roles in cancer chemoresistance in the PARPis- sensitive AsPCs, as compared to the PARPis- resistant AsPCs (see Additional file 7). Most of these genes/networks (comprising calpain, STAT1, ABCB1, LGALS8, CARD10, GST, LAMB1, PBX1, AHR, IFI16, the ATPase, the MAPK and MEK networks) were shown to be related to mechanisms of EOC chemoresistance, including association with advanced EOC stage and poor prognosis [73–85]. Interestingly, ABCB1 induction was also shown to define a common resistance mechanism in paclitaxel- and olaparib-resistant EOC cells [74, 86]. About a dozen of genes with previous shown implication in chemoresistance mechanisms in other cancer types, also displayed downregulation in the sensitive AsPCs. Thus, our data suggest that the PARPis-sensitive AsPCs could display enhanced sensitivity to other chemotherapeutic drugs, commonly applied in cancer (including EOC) therapy.

IPA canonical pathway analyses were also indicative of the modulation of different oncogenic pathways in PARPis-sensitive AsPCs versus resistant AsPCs, including antigen presentation, interferon, toll-like receptor, TGF-β, IL-6 and p38 MAPK, Rho family GTPases JNK, NGF and PDGF signaling (see Additional file 8).

In search of potential biomarkers to predict PARPis responsiveness, and based on our microarray data, we selected 24 genes based on their previously described relevant roles in tumorigenesis (including EOC tumorigenesis), EOC chemoresistance, and potential implications in DNA damage response and HR function (listed in Additional file 9). Four of these potential biomarkers (*C-MET, CDKN2A, N-cadherin and P-glyc/ABCB1*) displayed significantly lower expression levels in PARPis-sensitive AsPCs-matched HGSOC tumors. Among these, *C-MET*, a tyrosine kinase receptor, reported to be highly expressed in the four major EOC subtypes (high-grade serous, clear cell, mucinous, and endometrioid) [87–89] and is characterized as EOC prognostic marker and putative therapeutic target [90, 91]. *C-MET* inhibition enhances chemosensitivity of human EOC cells [92], and importantly, blocking *c-Met*-mediated PARP1 phosphorylation enhances anti-tumor effects of PARPis [93]. Indeed, it was recently shown that MET inhibitors function synergistically with PARPis in suppressing growth of triple-negative breast cancer cells and HGSOC cells [94]. The *CDKN2A (p16)* gene is a candidate tumor-suppressor gene in different cancer types [95], including EOC [96], where *p16* inactivation has been frequently associated with homozygous deletion or promoter hypermethylation [96, 97]. However, a number of reports were indicative for significantly higher p16 expression in HGSOC and undifferentiated ovarian carcinomas compared to low-grade and borderline serous carcinomas, suggesting that p16 overexpression may be implicated in the development of high-grade serous neoplasia within the ovary, possibly through inactivation of the retinoblastoma functional pathway [98, 99]. *N-cadherin* is a key EMT mediator in cancer, including EOC, as EOC cells undergoing EMT downregulate E-cadherin expression accompanied by increased expression of N-cadherin which promotes the interaction with endothelial and stromal components and thus increases cell migration and metastatic capacity [100]. Indeed, mesenchymal-type (N-cadherin +) cell lines easily create numerous widely-disseminated metastatic lesions in vivo, often accompanied by cancerous cachexia and ascites in mice [65, 67, 101]. Interestingly, it was recently shown that olaparib treatment can suppress in vitro cell migration and thus reduce the metastatic potential of different cancer cell lines, along with a decrease of the expression levels of *N-cadherin* and other EMT-related proteins, thus leading to suppression of EMT process [102, 103]. Furthermore, the role of *P-glycoprotein/ABCB1* drug transporter in EOC drug resistance, including resistance to PARPis therapy, was already discussed above.

Accordingly, three of the selected potential biomarkers (*SPRY2*, *E-cadherin* and *FANCF*) showed stronger expression in the sensitive AsPCs-matched HGSOC tumors. *SPRY2* (sprouty 2) belongs to the sprouty gene family, as members of this family function as inhibitors of the receptor tyrosine kinase-mediated activation of cellular signaling pathways [104]. *SPRY2* expression was shown to be significantly downregulated in human EOC, as low *SPRY2* expression significantly correlated with poorer progression-free and overall survival of EOC patients, suggestive for a role of *SPRY2* as an independent predictive EOC factor for survival and recurrence [105, 106], and as a possible EOC therapeutic target [105, 107]. Interestingly, a role of *SPRY2* in potentiating the *E-cadherin* expression in EOC cells has been demonstrated, which was associated with attenuated EOC cellular invasion and proliferation [105]. *E-cadherin* is key epithelial marker implicated in maintaining adherens junctions, which enables the cells to maintain epithelial phenotypes [108]. In general, tumor metastasis is associated with a loss of epithelial phenotype, concomitant with E-cadherin suppression and gain of *N-cadherin* expression [109–111]. *E-cadherin* frequently displays abundant expression in primary well-differentiated ovarian carcinomas [112–115], while loss or reduced *E-cadherin* expression is detected in ascites, late stage carcinomas and metastases [116–118], and is predictive of poor overall survival [65, 119]. *E-cadherin* expression was also shown to be markedly reduced in ascites-derived spheroids compared with adherent cells, accompanied by an up-regulation of *N-cadherin* and other mesenchymal EMT markers [120]. Similar to our data, a recent report was indicative for the enhancement of *E-cadherin* expression in EOC cells upon treatment with the PARP inhibitor PJ34, associated with decreased cellular proliferation and invasion due to the PJ34-mediated EMT attenuation [121]. However, the effect of PARPis on *E-cadherin* expression might be cancer type-specific, since PARPis treatment downregulated *E-cadherin* expression in small cell lung cancer (SCLC) cells, which could possibly explain the rapid development of therapeutic resistance in SCLC [122]. Similarly, the *FANCF* upregulation in the PARPis-sensitive AsPCs and their matched HGSOC tumors observed by us could be due to the development of resistance to the PARPis-treatment. Indeed, *FANCF* expression pattern was the only “inconsistent” result obtained during validation of our potential biomarkers since the members of the Fanconi anemia (FA) gene family, as part of the FA/BRCA pathway are involved in HR-mediated DNA repair which implicates their possible role in cell response to DNA-damaging agents in different tumor cells, including EOC tumors [123]. It has been shown that *FANCF* suppression due to promoter hypermethylation plays an important role in enhanced EOC occurrence and poor disease outcome [124, 125]. Moreover, shRNA-mediated *FANCF* silencing potentiated the cytotoxicity of the chemotherapeutic agents adriamycin and mitomycin-c in EOC cells [126, 127]. However, and as repeatedly stated, the mechanisms of PARPis resistance in EOC are multifactorial, the most common being restoration in HR and replication fork protection [128]. Interestingly, recent findings suggest that another FA family member—the Fanconi Anemia group D2 protein (*FANCD2*) can confer resistance to PARP inhibitors through replication fork stabilization, independent of HR dysfunction, or restoration [129]. Thus, further studies could be needed to more profoundly understand the putative mechanisms of *FANCF*-mediated PARPis resistance in EOC. Overall, the above described seven biomarkers could represent useful tools of potential benefits in predicting sensitivity of EOC patients to PARPis targeted therapy.

## Conclusion

We have developed a 3D (spheroid) functional assay to assess the sensitivity of two PARPis, niraparib and olaparib, in AsPCs derived from HGSOC patients. Most of the AsPCs examined displayed higher sensitivity upon treatment with niraparib as compared to olaparib. Global gene expression profiling of 6 PARPi-resistant and 6 PARPi-sensitive AsPCs identified 24 potential gene biomarkers associated with PARPis sensitivity/resistance. The differential protein expression of 7 selected biomarkers was consecutively confirmed by immunohistochemistry in the corresponding (matched) EOC tumor samples. Our 3D functional assay is relatively simple and can be carried out in a time frame compatible with its use as a tool to direct subsequent therapy. The application of this assay and the potential biomarkers with possible predictive significance to PARPis therapy of EOC patients now need testing in the setting of a clinical trial. Undoubtedly, the development of clinically feasible diagnostic assays and accurate biomarkers would optimize the efficacy of DNA repair targeted therapies and maximize their impact on cancer treatment.

## Supplementary information

**Additional file 1. **Antibodies used for Western Blots, immunohistochemistry and immunofluorescence. The Table includes list and detailed description of the antibodies used in the study, including their dilutions and incubation periods applied, for WB, IHC and IF.

**Additional file 2. **Primers used for qPCR. All primers used for qPCR experiments performed in the study. The table includes a list of the genes primers were designed for, the perimers primer melting temperature (Tm), and the design of the forward and reverse primers.

**Additional file 3**. Comparative analysis of PARPis-sensitive and resistant AsPCs, as examined in monolayer vs. 3D culture. (A) Total number of AsPCs determined as resistant or sensitive to treatment with the two PARPis olaparib and niraparib when grown in monolayer. Olaparib resistant AsPCs showed to be 36% higher in total number to niraparib resistant AsPCs, likewise niraparib sensitive AsPCs showed to be 36% higher in total number to olaparib sensitive AsPCs. (B) Total number of AsPCs determined as resistant or sensitive to treatment with the two PARPis olaparib and niraparib when treated in 3D. Olaparib resistant AsPCs showed to be 28% higher in total number to niraparib resistant AsPCs, likewise niraparib sensitive AsPCs showed to be 28% higher in total number to olaparib sensitive AsPCs.

**Additional file 4**. PARPis sensitive and resistant AsPCs present with different EMT features. (A) Western blot protein expression analysis of the two EMT markers, N-cadherin and E-cadherin in resistant (R) and sensitive (S) PARPis AsPCs. Actin was used as the loading control (n = 3). Histograms represent 6 resistant (R) and 6 sensitive (S) AsPCs, and the protein expression levels were normalized to actin. The two-tailed unpaired t-test was used for statistical analysis. All values were expressed as the means ± S.D. *p < 0.05 **p < 0.01 and ***p < 0.001 (B) Immunofluorescence analysis of the two EMT markers E-cadherin and N-cadherin in resistant (R) vs. sensitive (S) AsPCs. Scale bar = 20 µm.

**Additional file 5**. The effect of olaparib and niraparib on EMT in PARPis-resistant AsPCs. AsPCs were grown in monolayers for 48 h and then treated with either olaparib at a concentration of 50 μM for a period of 24 h, or niraparib at a concentration of 25 μM for a period of 24 h, as non-treated AsPCs were used as controls. Western blot protein expression analysis of the two EMT markers, (**A**) olaparib resistant and (**B**) niraparib resistant AsPCs. Actin was used as the loading control.

**Additional file 6**. Genes, differentially expressed between Sensitive (S) and resistant (R) AsPCs (≥ 1.5 fold, *p* ≤ 0.05). Table presenting the subset of differentially expressed genes that were selected by initial filtering on confidence at *p*-value ≤ 0.05, followed by filtering of expression level (≥ 1.5 fold). Using these selection criteria, the table lists 240 upregulated genes and 583 downregulated genes in the PARPis-sensitive AsPCs, as compared to the PARPis-resistant AsPCs.

**Additional file 7. **IPA network analysis of dynamic gene expression in PARPis-sensitive vs. PARPis-resistant AsPCs based on the 1.5-fold gene expression list obtained. The five top-scoring networks of up- and downregulated genes were merged and are displayed graphically as nodes (genes/gene products) and edges (the biological relationships between the nodes). Intensity of node color indicates the degree of upregulation (red) or downregulation (green). Nodes are displayed using various shapes that represent the functional class of the gene product (square, cytokine, vertical oval, transmembrane receptor, rectangle, nuclear receptor, diamond, enzyme, rhomboid, transporter, hexagon, translation factor, horizontal oval, transcription factor, circle, etc.). Edges are displayed with various labels that describe the nature of the relationship between the nodes: __ binding only, → acts on. Dotted edges represent indirect interaction. Highlighted nodes in purple represent genes that are implicated in EOC tumorigenesis.

**Additional file 8. **Comparative canonical pathway analysis for a dataset of differentially expressed genes (≥ 1.5-fold; *p* < 0.05) as evaluated in PARPIs-sensitive vs. PARPIs-resistant AsPCs. (A) Downregulated canonical pathways in the PARPIs-sensitive AsPCs, as compared to the PARPIs-resistant AsPCs; (B) upregulated canonical pathways in the PARPIs-sensitive AsPCs, as compared to the PARPIs-resistant AsPCs. Top functions that meet a Holm–Bonferroni multiple testing correction *p*-value of 0.05 are displayed.

**Additional file 9.** Quantitative PCR (qPCR) validation of the expression levels of 24 selected potential biomarkers, differentially expressed in PARPis-sensitive vs PARPis-resistant AsPCs. The microarray data-based differential expression levels of these 24 potential biomarkers were further confirmed by qPCR in AsPCs included in our test and validation sets. The relative copy number was calculated based on the target gene/18S ribosomal RNA ratio. Values more than or equal to 1 represent gene upregulation and less than 1 display gene downregulation.

**Additional file 10.** Original blots from Fig. 4, Additional file 4 and Additional file 5.

## Data Availability

The microarray data generated by this study have been deposited to the GEO database (https://www.ncbi.nlm.nih.gov/geo/) with Accession Number GSE149940.

## Abbreviations

HGSOC: High-grade serous ovarian cancer
PARPis: Poly(ADP-ribose) polymerase inhibitors
EOC: Epithelial ovarian cancer
HR: Homologous recombination
HRD: Homologous recombination deficiency
AsPCs: Ascites-derived primary cell cultures
MCAs: Multicellular aggregates
3D: Three dimensional
EMT: Epithelial to mesenchymal transition
FDA: Food and Drug Administration
EMA: European Medicines Agency
NGS: Next generation sequencing
LOH: Loss of heterozygosity
TAI: Telomeric allelic imbalance
LST: Large-scale state transitions
qPCR: Quantitative PCR
TMAs: Tissue micro-arrays
IHC: Immunohistochemistry
IPA software: Ingenuity Pathway Analysis software
FA: Fanconi anemia
shRNA: Short hairpin RNA
