# Supplementary material for: Development of a 3D functional assay and identification of biomarkers, predictive for response of high-grade serous ovarian cancer (HGSOC) patients to poly-ADP ribose polymerase inhibitors (PARPis): targeted therapy
Source: J Transl Med. 2020 Nov 19;18:439. doi: 10.1186/s12967-020-02613-4 (PMC7678187; doi:10.1186/s12967-020-02613-4)
Supplement: Supplementary file 1 — Additional file 1. Antibodies used for Western Blots, immunohistochemistry and immunofluorescence. The Table includes list and detailed description of the antibodies used in the study, including their dilutions and incubation periods applied, for WB, IHC and IF. [file 12967_2020_2613_MOESM1_ESM.docx]

Additional file 1: Antibodies used for Western Blots, immunohistochemistry and immunofluorescence.

| **Antibody** | **Species** | **Company** | **Dilution WB** | **Dilution IHC** | **Dilution**  **IF** | **Incubation WB** | **Incubation IHC** | **Incubation IF** |
| --- | --- | --- | --- | --- | --- | --- | --- | --- |
| N-cadherin | Mouse | Abcam | N/A | N/A | 1:250 | N/A | N/A | 4° C overnight |
| E-cadherin | Rabbit | Abcam | N/A | N/A | 1:250 | N/A | N/A | 4° C overnight |
| N-cadherin | Mouse | Santa-Cruz Biotechnology | 1:1000 | 1:100 | N/A | 4° C overnight | 4° C overnight | N/A |
| E-cadherin | Mouse | Santa-Cruz Biotechnology | 1:1000 | 1:100 | N/A | 4° C overnight | 4° C overnight | N/A |
| beta-actin | Mouse | Santa-Cruz Biotechnology | 1:1000 | N/A | N/A | 4° C overnight | N/A | N/A |
| H2AX | Mouse | Millipore | N/A | N/A | 1:500 | N/A | N/A | 4° C overnight |
| C-Met | Mouse | LSBio | N/A | 1:150 |  | N/A | 4° C overnight |  |
| CDKN2A | Rabbit | Abcam | N/A | 1:100 |  | N/A | 4° C overnight |  |
| P-glyc (ABCB1) | Rabbit | Abcam | N/A | 1:100 |  | N/A | 4° C overnight |  |
| FANCF | Rabbit | LSBio | N/A | 1:100 |  | N/A | 4° C overnight |  |
| SPRY2 | Mouse | Abcam | N/A | 1:75 |  | N/A | 4° C overnight |  |

N/A, not applicable; WB, Western blotting; IHC, immunohistochemistry; IF, immunofluorescence.
