# Supplementary material for: Development of a 3D functional assay and identification of biomarkers, predictive for response of high-grade serous ovarian cancer (HGSOC) patients to poly-ADP ribose polymerase inhibitors (PARPis): targeted therapy
Source: J Transl Med. 2020 Nov 19;18:439. doi: 10.1186/s12967-020-02613-4 (PMC7678187; doi:10.1186/s12967-020-02613-4)
Supplement: Supplementary file 2 — Additional file 2. Primers used for qPCR. All primers used for qPCR experiments performed in the study. The table includes a list of the genes primers were designed for, the perimers primer melting temperature (Tm), and the design of the forward and reverse primers. [file 12967_2020_2613_MOESM2_ESM.docx]

Additional file 2. Primers used for qPCR

| Gene | Tm(°C) | Length(bp) | Forward | Reverse |
| --- | --- | --- | --- | --- |
| **qPCR primers** | | | | |
| JAG1 | 60 | 190 | GACTCATCAGCCGTGTCTCA | TGGGGAACACTCACACTCAA |
| ANXA3 | 60 | 159 | CGCAGTTTGTTCGCAGTTTA | GCTGTGCATTTGACCTCTCA |
| MET | 60 | 201 | CAGGCAGTGCAGCATGTAGT | GATGATTCCCTCGGTCAGAA |
| PTGER4 | 60 | 210 | AAGCTGGGACTCGTCTTTGA | GCTTTCACCTTGTCCTGCTC |
| RTKN2 | 60 | 216 | AGAAGATGCTGCAGGGAAAA | ACTCTGAGGGCACAACTGCT |
| CASP1 | 60 | 160 | GCTTTCTGCTCTTCCACACC | CATCTGGCTGCTCAAATGAA |
| ANXA1 | 60 | 203 | GCAGGCCTGGTTTATTGAAA | GCTGTGCATTGTTTCGCTTA |
| IL18 | 60 | 227 | GGTTCTCTGGAGGCTGAGTG | CCAGGAGCAAAACACTGACA |
| PARP9 | 60 | 200 | CAATGGTCGTGAACAACCTG | CAACTGGGACCGTTGAAACT |
| BMP4 | 60 | 180 | TGAGCCTTTCCAGCAAGTTT | CTTCCCCGTCTCAGGTATCA |
| PARP14 | 60 | 244 | GACAGGCTGGTCTCGAACTC | CATCAAACGCTAGGCTGTCA |
| ABCB1 | 60 | 202 | GCTCCTGACTATGCCAAAGC | TCTTCACCTCCAGGCTCAGT |
| CDK2NA | 60 | 231 | ATATGCCTTCCCCCACTACC | CCCCTGAGCTTCCCTAGTTC |
| PRR15 | 60 | 228 | CCCCCAAACCAGACAAGTTA | TTTGGGGTAGTGCTGGGTAG |
| NBEA | 60 | 213 | GTTGGCTTGATGGAACAGGT | CCCAAAATGGCAAATAATGG |
| ZFP82  HMGA2 | 60 | 188 | ATGTGTCTGCCCCCTCTATG | AAGCCAAGATCTGCGACAGT |
|  | 60 | 208 | CTCTCCACCCTTTCAATCCA | CACACCACCCACAGAAGATG |
| TCF4  NDN | 60 | 203 | TTATGAATCGCAGACGCAAG | GCACAAATGCATTGTTCCAC |
|  | 60 | 164 | GAAGAAGCACTCCACCTTCG | CCATGATTTGCATCTTGGTG |
| SPRY2  FANCF | 60 | 191 | CTAAGCCTGCTGGAGTGACC | GTGTTTCGGATGGCTCTGAT |
|  | 60 | 190 | GCTAGTCCACTGGCTTCTGG | GGACTCAGTTCCAACCCAAA |
| DDR2  FGFR1 | 60 | 183 | CCACTATGCAGAGGCTGACA | CAGAGATGAACCTCCCCAAA |
|  | 60 | 188 | ATGTGTCTGCCCCCTCTATG | AAGCCAAGATCTGCGACAGT |
| 18S | 60 | 119 | AACCCGTTGAACCCCATT | CCATCCAATCGGTAGTAGCG |
