# Supplementary figures and images for: Development of a 3D functional assay and identification of biomarkers, predictive for response of high-grade serous ovarian cancer (HGSOC) patients to poly-ADP ribose polymerase inhibitors (PARPis): targeted therapy
Source: J Transl Med. 2020 Nov 19;18:439. doi: 10.1186/s12967-020-02613-4 (PMC7678187; doi:10.1186/s12967-020-02613-4)

## Slide 1
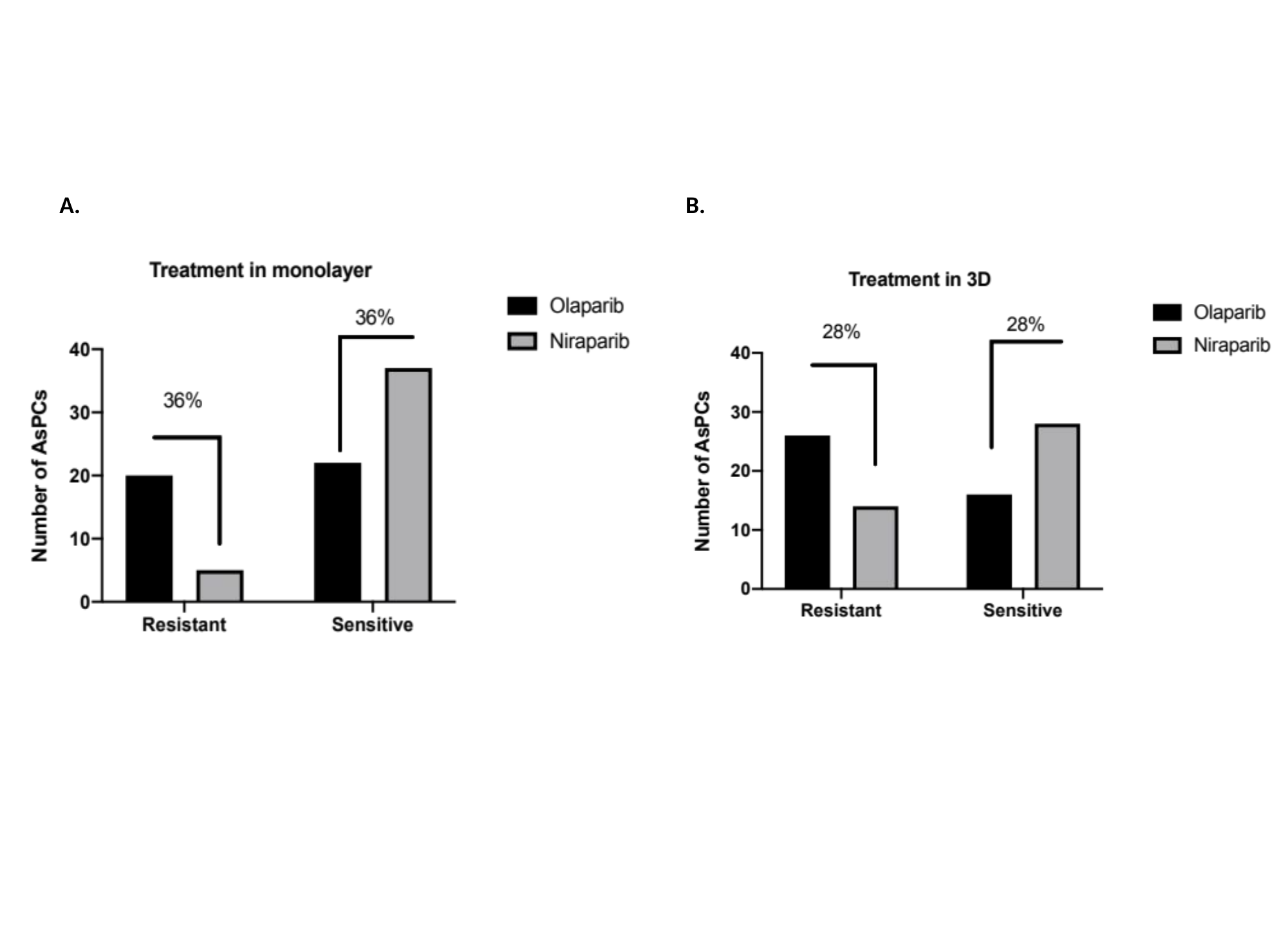

A.
B.

Supplement: Supplementary file 3 — Additional file 3. Comparative analysis of PARPis-sensitive and resistant AsPCs, as examined in monolayer vs. 3D culture. (A) Total number of AsPCs determined as resistant or sensitive to treatment with the two PARPis olaparib and niraparib when grown in monolayer. Olaparib resistant AsPCs showed to be 36% higher in total number to niraparib resistant AsPCs, likewise niraparib sensitive AsPCs showed to be 36% higher in total number to olaparib sensitive AsPCs. (B) Total number of AsPCs determined as resistant or sensitive to treatment with the two PARPis olaparib and niraparib when treated in 3D. Olaparib resistant AsPCs showed to be 28% higher in total number to niraparib resistant AsPCs, likewise niraparib sensitive AsPCs showed to be 28% higher in total number to olaparib sensitive AsPCs. [file 12967_2020_2613_MOESM3_ESM.pptx]

## Slide 1
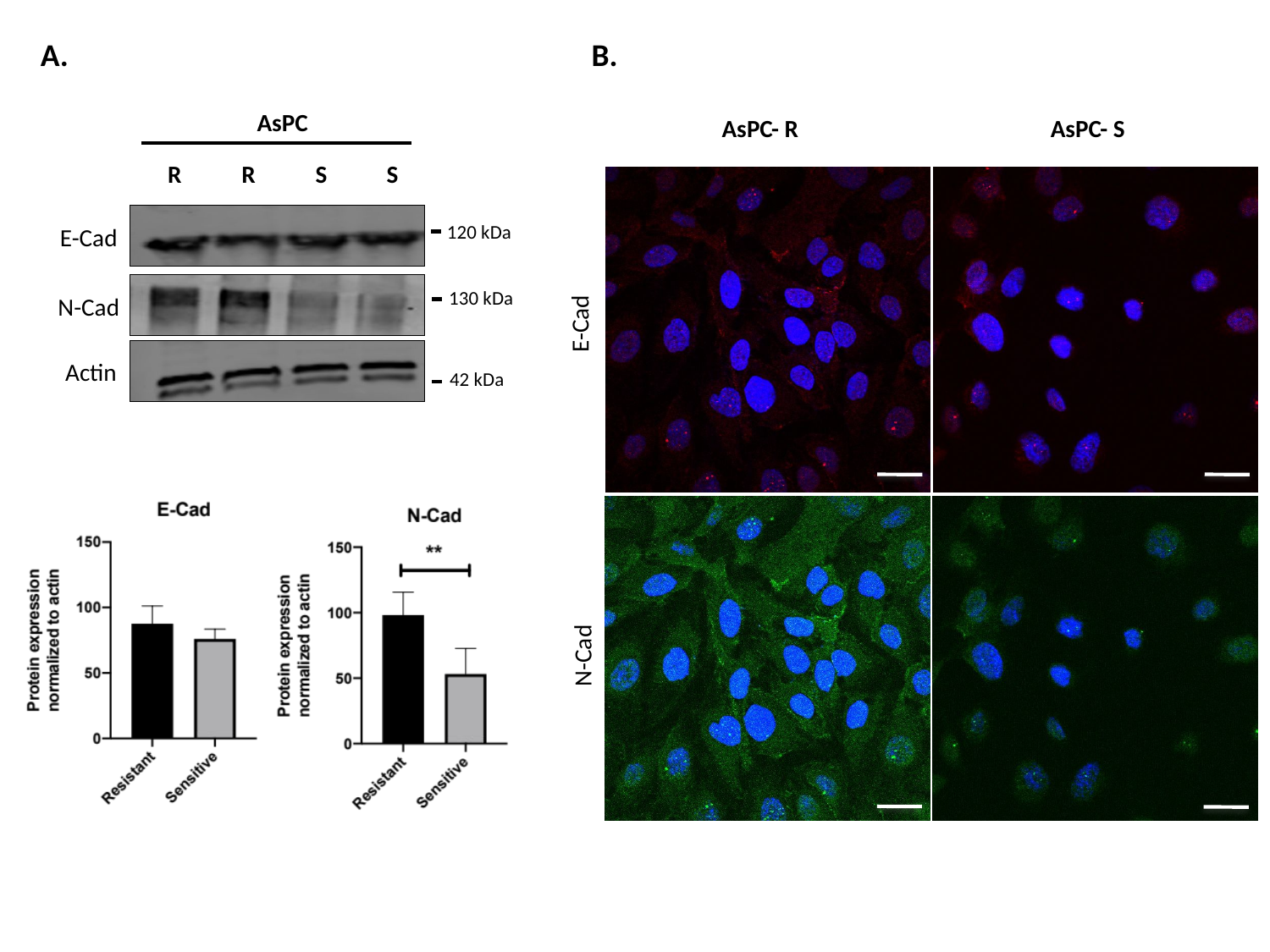

A.
B.
AsPC
AsPC- R
AsPC- S
R
R
S
S
120 kDa
E-Cad
130 kDa
N-Cad
E-Cad
Actin
42 kDa
N-Cad

Supplement: Supplementary file 4 — Additional file 4. PARPis sensitive and resistant AsPCs present with different EMT features. (A) Western blot protein expression analysis of the two EMT markers, N-cadherin and E-cadherin in resistant (R) and sensitive (S) PARPis AsPCs. Actin was used as the loading control (n = 3). Histograms represent 6 resistant (R) and 6 sensitive (S) AsPCs, and the protein expression levels were normalized to actin. The two-tailed unpaired t-test was used for statistical analysis. All values were expressed as the means ± S.D. *p < 0.05 **p < 0.01 and ***p < 0.001 (B) Immunofluorescence analysis of the two EMT markers E-cadherin and N-cadherin in resistant (R) vs. sensitive (S) AsPCs. Scale bar = 20 µm. [file 12967_2020_2613_MOESM4_ESM.pptx]

## Slide 1
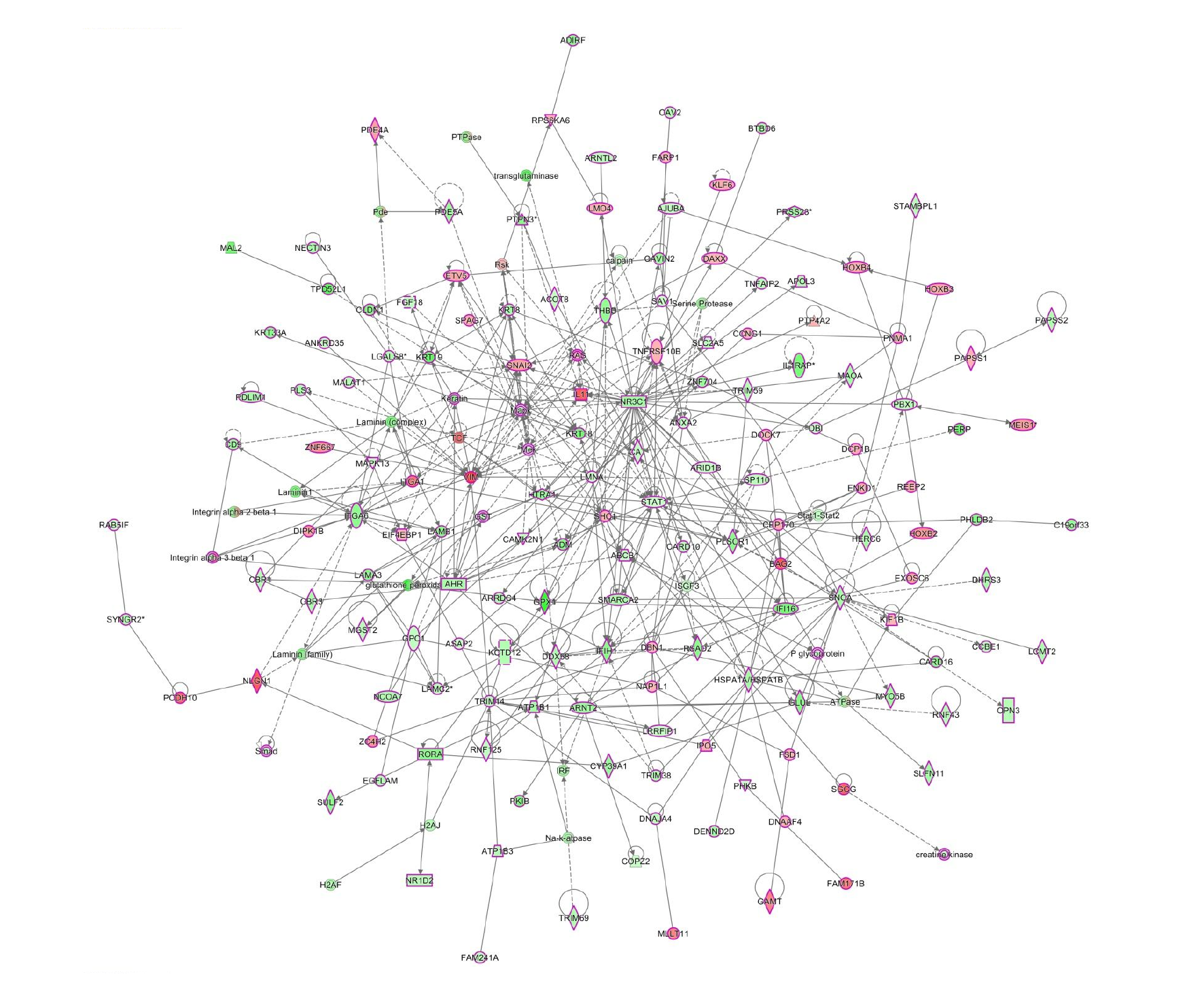

Supplement: Supplementary file 7 — Additional file 7. IPA network analysis of dynamic gene expression in PARPis-sensitive vs. PARPis-resistant AsPCs based on the 1.5-fold gene expression list obtained. The five top-scoring networks of up- and downregulated genes were merged and are displayed graphically as nodes (genes/gene products) and edges (the biological relationships between the nodes). Intensity of node color indicates the degree of upregulation (red) or downregulation (green). Nodes are displayed using various shapes that represent the functional class of the gene product (square, cytokine, vertical oval, transmembrane receptor, rectangle, nuclear receptor, diamond, enzyme, rhomboid, transporter, hexagon, translation factor, horizontal oval, transcription factor, circle, etc.). Edges are displayed with various labels that describe the nature of the relationship between the nodes: __ binding only, → acts on. Dotted edges represent indirect interaction. Highlighted nodes in purple represent genes that are implicated in EOC tumorigenesis. [file 12967_2020_2613_MOESM7_ESM.pptx]

## Slide 1
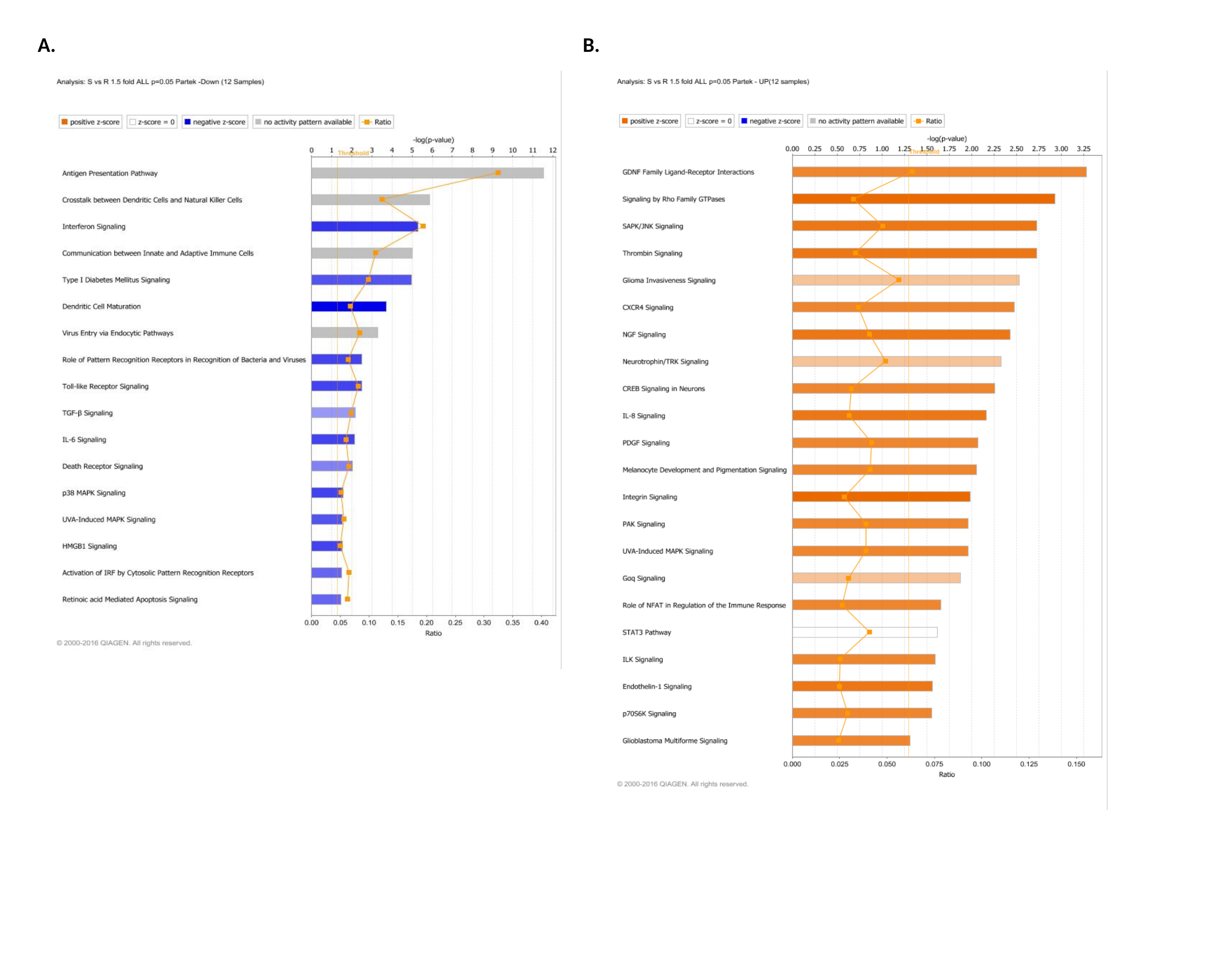

A.
B.

Supplement: Supplementary file 8 — Additional file 8. Comparative canonical pathway analysis for a dataset of differentially expressed genes (≥ 1.5-fold; p < 0.05) as evaluated in PARPIs-sensitive vs. PARPIs-resistant AsPCs. (A) Downregulated canonical pathways in the PARPIs-sensitive AsPCs, as compared to the PARPIs-resistant AsPCs; (B) upregulated canonical pathways in the PARPIs-sensitive AsPCs, as compared to the PARPIs-resistant AsPCs. Top functions that meet a Holm–Bonferroni multiple testing correction p-value of 0.05 are displayed. [file 12967_2020_2613_MOESM8_ESM.pptx]

## Slide 1
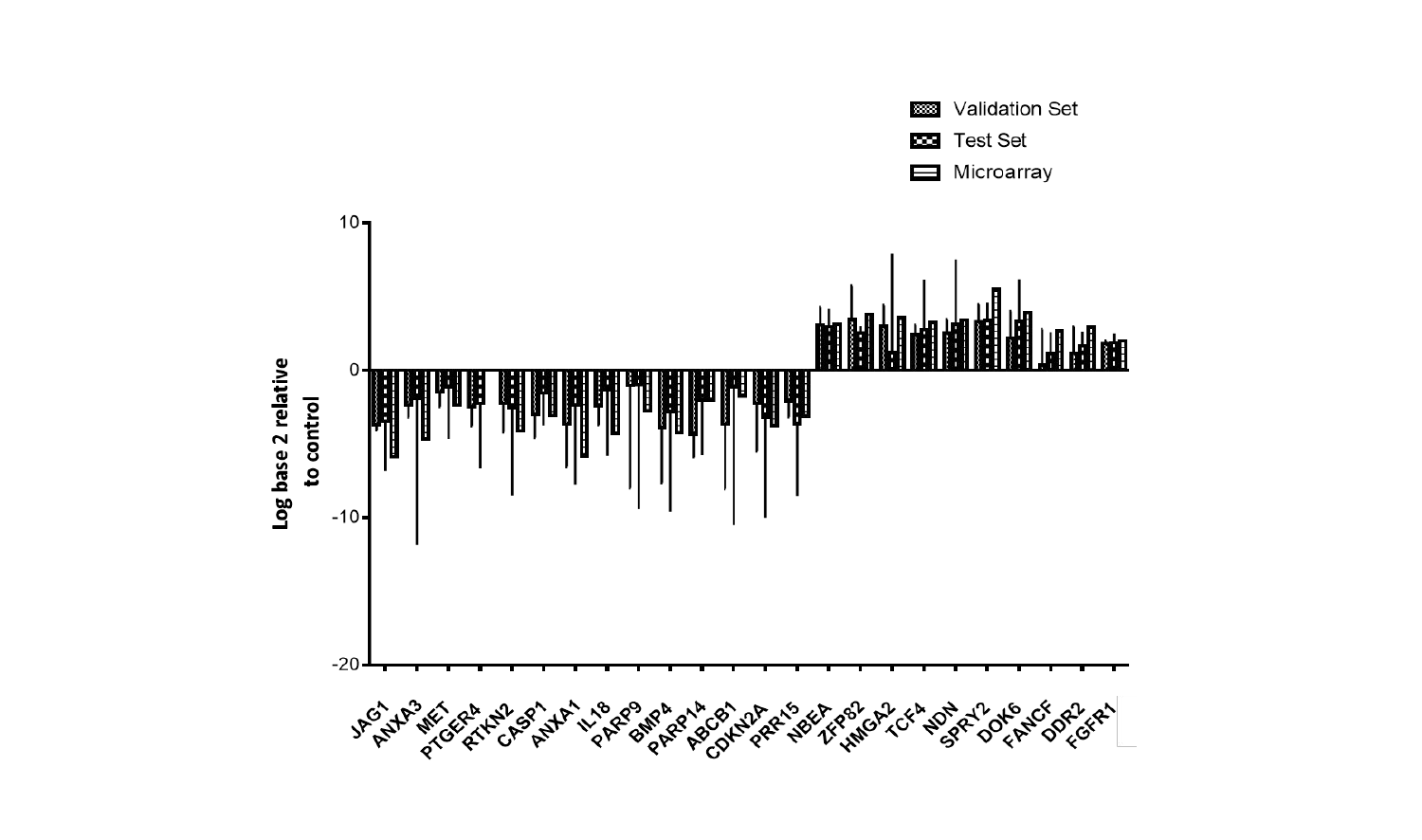

Supplement: Supplementary file 9 — Additional file 9. Quantitative PCR (qPCR) validation of the expression levels of 24 selected potential biomarkers, differentially expressed in PARPis-sensitive vs PARPis-resistant AsPCs. The microarray data-based differential expression levels of these 24 potential biomarkers were further confirmed by qPCR in AsPCs included in our test and validation sets. The relative copy number was calculated based on the target gene/18S ribosomal RNA ratio. Values more than or equal to 1 represent gene upregulation and less than 1 display gene downregulation. [file 12967_2020_2613_MOESM9_ESM.pptx]
