# Supplementary material for: Development of a 3D functional assay and identification of biomarkers, predictive for response of high-grade serous ovarian cancer (HGSOC) patients to poly-ADP ribose polymerase inhibitors (PARPis): targeted therapy
Source: J Transl Med. 2020 Nov 19;18:439. doi: 10.1186/s12967-020-02613-4 (PMC7678187; doi:10.1186/s12967-020-02613-4)
Supplement: Supplementary file 5 — Additional file 5. The effect of olaparib and niraparib on EMT in PARPis-resistant AsPCs. AsPCs were grown in monolayers for 48 h and then treated with either olaparib at a concentration of 50 μM for a period of 24 h, or niraparib at a concentration of 25 μM for a period of 24 h, as non-treated AsPCs were used as controls. Western blot protein expression analysis of the two EMT markers, (A) olaparib resistant and (B) niraparib resistant AsPCs. Actin was used as the loading control. [file 12967_2020_2613_MOESM5_ESM.pptx]

## Slide 1
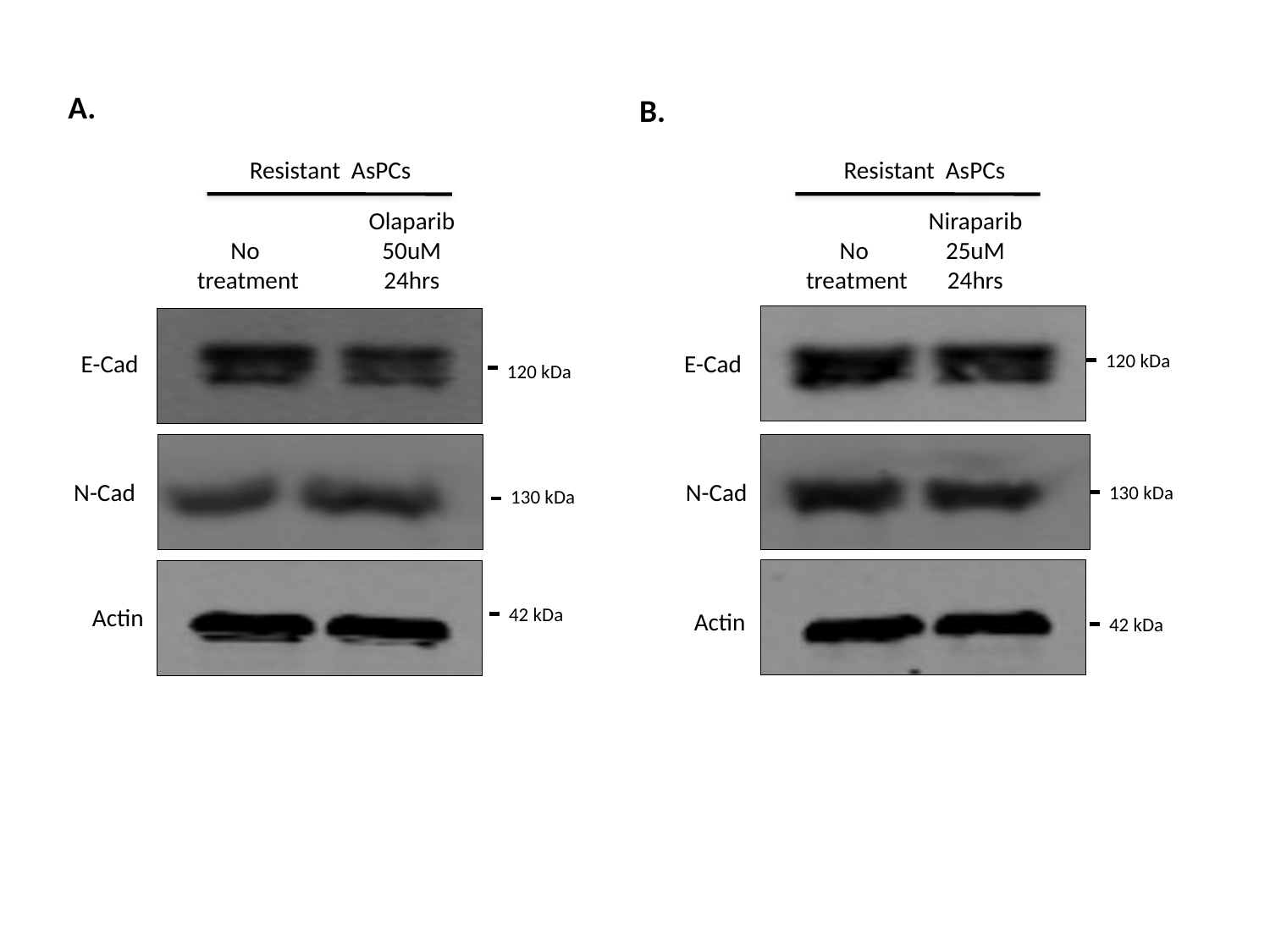

A.
B.
Resistant AsPCs
Resistant AsPCs
Olaparib
50uM
24hrs
Niraparib
25uM
24hrs
No
 treatment
No
 treatment
E-Cad
E-Cad
120 kDa
120 kDa
N-Cad
N-Cad
130 kDa
130 kDa
Actin
42 kDa
Actin
42 kDa
