# Supplementary material for: Development of a 3D functional assay and identification of biomarkers, predictive for response of high-grade serous ovarian cancer (HGSOC) patients to poly-ADP ribose polymerase inhibitors (PARPis): targeted therapy
Source: J Transl Med. 2020 Nov 19;18:439. doi: 10.1186/s12967-020-02613-4 (PMC7678187; doi:10.1186/s12967-020-02613-4)
Supplement: Supplementary file 10 — Additional file 10. Original blots from Fig. 4, Additional file 4 and Additional file 5. [file 12967_2020_2613_MOESM10_ESM.pptx]

## Slide 1
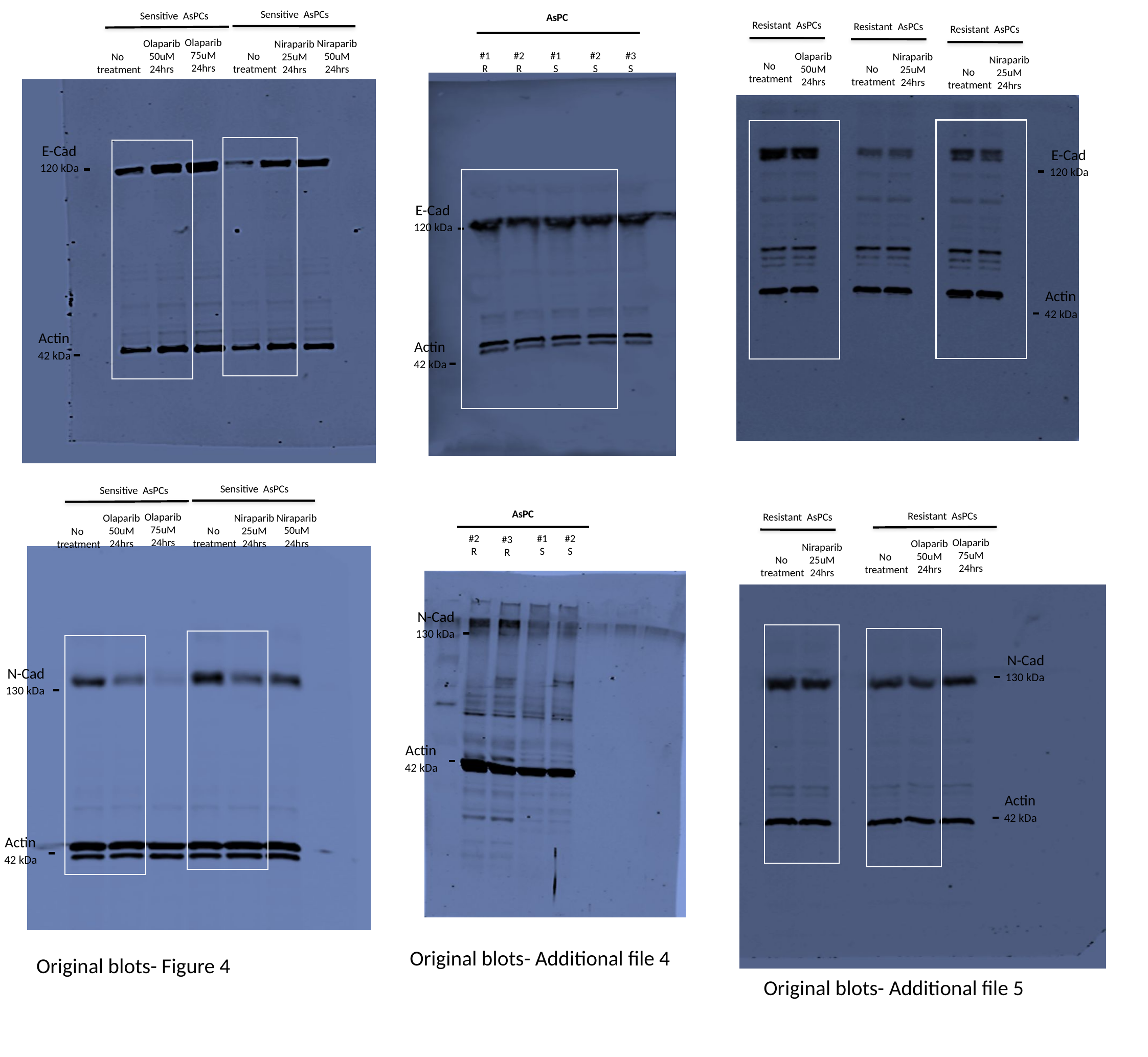

Sensitive AsPCs
Sensitive AsPCs
AsPC
Resistant AsPCs
Resistant AsPCs
Resistant AsPCs
Olaparib
75uM
24hrs
Niraparib
50uM
24hrs
Olaparib
50uM
24hrs
Niraparib
25uM
24hrs
#1
R
#2
R
#1
S
#2
S
#3
S
No
 treatment
Olaparib
50uM
24hrs
Niraparib
25uM
24hrs
No
 treatment
Niraparib
25uM
24hrs
No
 treatment
No
 treatment
No
 treatment
E-Cad
E-Cad
120 kDa
120 kDa
E-Cad
120 kDa
Actin
42 kDa
Actin
Actin
42 kDa
42 kDa
Sensitive AsPCs
Sensitive AsPCs
AsPC
Resistant AsPCs
Resistant AsPCs
Olaparib
75uM
24hrs
Niraparib
50uM
24hrs
Olaparib
50uM
24hrs
Niraparib
25uM
24hrs
No
 treatment
No
 treatment
#2
R
#1
S
#2
S
#3
R
Olaparib
75uM
24hrs
Olaparib
50uM
24hrs
Niraparib
25uM
24hrs
No
 treatment
No
 treatment
N-Cad
130 kDa
N-Cad
N-Cad
130 kDa
130 kDa
Actin
42 kDa
Actin
42 kDa
Actin
42 kDa
Original blots- Additional file 4
Original blots- Figure 4
Original blots- Additional file 5
